# Data‐driven discovery of associations between prescribed drugs and dementia risk: A systematic review

**DOI:** 10.1002/trc2.70037

**Published:** 2025-01-21

**Authors:** Benjamin R. Underwood, Ilianna Lourida, Jessica Gong, Stefano Tamburin, Eugene Yee Hing Tang, Emad Sidhom, Xin You Tai, Matthew J. Betts, Janice M. Ranson, Margarita Zachariou, Olajide E. Olaleye, Saswati Das, Neil P. Oxtoby, Shanquan Chen, David J. Llewellyn, for the Deep Dementia Phenotyping (DEMON) Network

**Affiliations:** ^1^ Department of Psychiatry and Cambridgeshire and Peterborough NHS Foundation Trust, Windsor Unit Fulbourn Hospital Cambridge University of Cambridge Cambridge UK; ^2^ NIHR Applied Research Collaboration South West (PenARC) University of Exeter Exeter UK; ^3^ Department of Epidemiology and Public Health University College London London UK; ^4^ Department of Neurosciences, Biomedicine and Movement Sciences University of Verona Verona Italy; ^5^ Population Health Sciences Institute Newcastle University Newcastle UK; ^6^ Depatment of Clinical neurosciences University of Cambridge, and Cambridge and Peterborough NHS Foundation Trust, Windsor Unit, Fulbourn Hospital Cambridge UK; ^7^ Nuffield Department of Clinical Neurosciences University of Oxford Oxford UK; ^8^ Institute of Cognitive Neurology and Dementia Research Otto‐von‐Guericke University Magdeburg Magdeburg Germany; ^9^ German Center for Neurodegenerative Diseases (DZNE) Magdeburg Germany; ^10^ Department of Health and Community Sciences, Medical School University of Exeter Exeter UK; ^11^ Bioinformatics Department The Cyprus Institute of Neurology and Genetics Nicosia Cyprus; ^12^ Department of Laboratory Medicine and Pathology Mayo Clinic Rochester Minnesota USA; ^13^ Atal Bihari Vajpayee Institute of Medical Sciences & Dr Ram Manohar Lohia Hospital New Delhi India; ^14^ UCL Centre for Medical Image Computing Department of Computer Science University College London London UK; ^15^ International Centre for Evidence in Disability London School of Hygiene & Tropical Medicine London UK; ^16^ The Alan Turing Institute London UK

**Keywords:** medications, drug repurposing, electronic health records, machine learning, pharmacoepidemiology, dementia, Alzheimer's disease

## Abstract

**Abstract:**

Recent clinical trials on slowing dementia progression have led to renewed focus on finding safer, more effective treatments. One approach to identify plausible candidates is to assess whether existing medications for other conditions may affect dementia risk. We conducted a systematic review to identify studies adopting a data‐driven approach to investigate the association between a wide range of prescribed medications and dementia risk. We included 14 studies using administrative or medical records data for more than 130 million individuals and 1 million dementia cases. Despite inconsistencies in identifying specific drugs that may modify Alzheimer's or dementia risk, some themes emerged for drug classes with biological plausibility. Antimicrobials, vaccinations, and anti‐inflammatories were associated with reduced risk, while diabetes drugs, vitamins and supplements, and antipsychotics were associated with increased risk. We found conflicting evidence for antihypertensives and antidepressants. Drug repurposing for use in dementia is an urgent priority. Our findings offer a basis for prioritizing candidates and exploring underlying mechanisms.

**Highlights:**

· We present a systematic review of studies reporting association between drugs prescribed for other conditions and risk of dementia including 139 million people and 1 million cases of dementia.
· Our work supports some previously reported associations, for example, showing decreased risk of dementia with drugs to treat inflammatory disease and increased risk with antipsychotic treatment.
· Antimicrobial treatment was perhaps more surprisingly associated with decreased risk, supportive of recent increased interest in this potential therapeutic avenue.
· Our work should help prioritize drugs for entry into adaptive platform trials in Alzheimer's disease and will serve as a useful resource for those investigating drugs or classes of drugs and risk of dementia.

## 1 INTRODUCTION

Dementia is a leading cause of morbidity and mortality and has an estimated worldwide economic cost in excess of 1 trillion dollars.^1^ It can lead to profound distress in the individual and in those caring for them. As such, dementia represents one of the most important challenges in medicine and public health. Current medical treatments for dementia are symptomatic and have a modest effect. Despite intensive efforts, trial results of disease‐modifying drugs have historically been largely disappointing. Any disease‐modifying drug would need only a relatively modest effect to have a significant impact; delaying the onset of Alzheimer's disease (AD) by 5 years would translate into lower prevalence and its associated costs by 40%.^2^ The recent findings that two new drugs, lecanemab and donanemab, reduce amyloid plaque levels in early symptomatic AD and result in statistically significant clinical benefits are a momentous step in the field.^3^, ^4^ However, these drugs target a single pathway in a complex condition, carry a significant risk of severe side effects, and there is wide consensus that multiple approaches are likely to be needed to provide maximally effective treatment.^5^, ^6^ An increasing number of pathological mechanisms have been identified and these have been targeted by both novel and repurposed drugs. Importantly, many of these mechanisms, for example, protein misfolding, production, and degradation, are common to many of the underlying causes of dementia^7^ and therefore any treatment targeting a common mechanism may have benefit in several different conditions.

Once a pathogenic pathway is identified, generic drugs can be screened to find those that act on the pathway and be tested in animal models of disease. Associations can then be sought from large clinical databases to examine if these prespecified drugs are associated with altered incidence of dementia or individual diseases leading to dementia. Examples of this approach include assessing medications which may act on specific pathways such as inflammation or the unfolded protein response as well as drugs with pleiotropic proposed mechanisms.^8^, ^9^, ^10^

Examining risk reduction associated with already prescribed medications can complement the traditional drug discovery approach. Currently prescribed drugs may interact with dementia‐related pathophysiological pathways by way of mechanisms unrelated to their original therapeutic indication.^8^ For example, some but not all, drug treatments for diabetes have been associated with reduced dementia risk suggesting that any effect may be separate to the ability to lower blood glucose.^11^ Other drug classes, for example benzodiazepines, have been associated with increased risk of dementia^12^ although more recent studies suggest that the role of benzodiazepines in dementia risk is questionable.^13^

Available evidence is primarily based on large prospective cohort studies (eg, UK Biobank) or retrospective studies studying associations between specific drugs or classes and dementia incidence. An alternative approach is to utilize large clinical datasets and examine all drugs in current use for associations with dementia risk. The increasing availability of routinely collected data such as electronic health records (EHRs) and administrative health claims data, along with the application of more sophisticated methodological approaches, allow the study of associations between hundreds of drugs with multiple outcomes in millions of patients.  This approach has been adopted to identify individual drugs which have then been investigated in the lab to understand their potential mechanism, a reversal of the usual approach.^14^ Other studies have used routinely collected data to generate drug repurposing hypotheses or develop dementia risk prediction models. Some of these have identified specific medications or drug classes as important predictors of dementia risk, sometimes with conflicting results (eg, statins, antibiotics, antipsychotics).^15^, ^16^, ^17^

We conducted a systematic review to identify and summarize studies adopting a data‐driven approach to investigate the association between prescribed medications and dementia risk. Though this methodology excludes some high‐quality papers examining specific drugs or groups of drugs, it has the advantage of minimizing publication bias (only positive associations with hypothesized drugs being reported), serves as a complementary approach to attempt to replicate previously reported findings using alternative designs and, crucially, includes all currently prescribed drugs, which allows the potential for identifying drugs which might alter risk but which have not previously been the focus of research. Studies investigating many medications are liable to false positive findings. Each paper allowed for this in their original publication. However, one of our motivations for completing this review was to see if findings replicated in different databases using different techniques. If associations were true positives one would expect them to be identified in more than one paper taking this approach. Our aim was to identify consistent patterns of individual or classes of drugs which alter risk of a dementia diagnosis to support potential candidates for drug repurposing and inform risk reduction and prevention strategies.

## 2 METHODS

This systematic review was conducted following the general principles published by the National Health Service (NHS) Centre for Reviews and Dissemination (CRD)^18^ and is reported following the Preferred Reporting Items for Systematic reviews and Meta‐Analyses (PRISMA) reporting guideline.^19^ A protocol was developed following consultation with topic and methods experts and is registered with PROSPERO (PROSPERO 2022: CRD42022359187).

### 2.1 Study identification

A comprehensive search strategy was developed by the review team using a combination of subject headings (MeSH terms) and free‐text terms to cover the prescribed mediation, dementia, and EHRs fields. Literature searches were conducted using the following databases from inception through August 5, 2022: MEDLINE, Embase, and PsycINFO via the Ovid interface. Details of the searches can be found in Table S1. No language or methodological filters were applied in searching. Reference lists of included papers (backward citation searching) and citations of included papers (forward citation searching) were searched using the citation chasing tool “citationchaser” (https://estech.shinyapps.io/citationchaser/).^20^ Updated database searches were performed on August 10, 2023 using the same search methods narrowing the searches to August 2022 onwards.

### 2.2 Eligibility criteria

We were interested in peer‐reviewed studies following a data‐driven rather than hypothesis‐driven approach investigating the association between prescribed medication use and risk of developing dementia, where we defined data‐driven as “an exploratory approach that analyzes large datasets to extract insights and patterns by applying analytical techniques and modes of reasoning.” Specific search terms are included in supplementary data. Therefore, studies driven by a priori hypotheses, examining associations between *prespecified* drugs or drug classes (eg, antihypertensives, antipsychotics, acetylcholinesterase inhibitors, etc.) and dementia risk were excluded. Studies were included if they examined the association between prescribed medication use (or several potential predictors at least one of which being medication use) in adults and dementia incidence diagnosed according to standardized criteria, including all‐cause dementia and subtypes (eg, AD, vascular dementia, Lewy body disease, dementia in Parkinson's disease). We did not include preprints or other grey literature. We anticipated that eligible studies would use health records data from inpatient and outpatient settings; however, all settings and study designs were considered, providing they met the inclusion/exclusion criteria. Reviews, editorials, commentaries, protocols, conference abstracts, and letters to the editor were excluded. Backwards and forwards citation chasing was used to identify additional relevant studies.

### 2.3 Study selection

Search results were downloaded to Endnote version X9 (Clarivate, Philadelphia, PA), deduplicated and imported to the Rayyan reference management software^21^ for screening. Titles and abstracts were screened for relevance independently by pairs of reviewers (I.L. and E.Y.H.T., J.G., S.T., E.S., X.Y.T., J.M.R.). Disagreements were resolved by discussion between reviewers or with the involvement of a third reviewer (M.J.B.) where necessary. The full text of potentially relevant articles was retrieved and screened in the same way (I.L. and E.Y.H.T., J.G., J.M.R., M.Z., O.E.O., S.D.) using the predefined inclusion and exclusion criteria. Disagreements were resolved with the involvement of a third reviewer (B.R.U.).

### 2.4 Data collection and study quality assessment

Details on country, study design and aim, type of data used, sample size, number of medications and classification system, predictors, features or covariates used in models, outcome and method of assessment, time window/follow‐up duration, statistical analysis, and machine learning (ML) methods applied, prediction performance metrics for ML studies, and results related to prescribed medication and dementia risk were recorded for each study. Data were extracted by one reviewer and checked by a second using a predefined template in an Excel spreadsheet. Authors of four studies were contacted for clarification or additional data.

The quality of included studies was assessed by one reviewer (I.L., N.P.O., or S.C.) and checked by a second (I.L., N.P.O., or S.C.). In an amendment to the published protocol, risk of bias was assessed by the Prediction model Risk Of Bias ASsessment Tool (PROBAST)^22^ or the Joanna Briggs Institute (JBI) critical appraisal checklist tools^23^ depending on the purpose of the data‐driven approach of studies. PROBAST is designed to evaluate risk of bias and concerns regarding the applicability of prediction model studies and contains 20 questions under four domains: participants, predictors, outcome, and analysis. The overall risk of bias in the prediction models was judged as “low,” “high,” or “unclear” according to the PROBAST checklist.^22^ The JBI tools used contain 10 questions considering sample selection, assessment of exposure and outcome measures, statistical analysis, and strategies to deal with confounders and loss to follow‐up.^23^ The JBI appraisal checklist does not apply the overall risk of bias assessments. Nevertheless, we matched the JBI questions to the four PROBAST domains and provided overall assessments for descriptive purposes.

### 2.5 Data synthesis

Our focus is on synthesizing reported information regarding the association of prescribed medication with dementia risk. While some of the identified studies examined that specific relationship, others investigated a larger set of variables or features (including medications) which translated into a range of methodological approaches and formats in which results are reported, and this precluded formal pooling of the data. Therefore, data were tabulated, grouped according to purpose of data‐driven approach, direction of association, and outcome, and findings were discussed narratively. An additional sub‐analysis was conducted where the most comprehensive medication‐wide study by Wilkinson et al.^24^ was used as the base for comparisons to identify the medications significantly associated with dementia risk. Not only was this study comprehensive but it uses a cohort where follow‐up is from the 60th birthday to dementia diagnosis, a median of 17 years later, and therefore is more likely to reflect medications given in any potential prodromal period. First, we identified medications within the Wilkinson study with associations where *p*‐value < 0.001 for overall dementia risk and for 5‐year sensitivity analyses (ie, where participants first received a drug less than 5 years before dementia diagnosis were excluded). We then mapped these medications to (1) the findings of the other two medication‐wide studies, and (2) the remaining studies included in the review.

## 3. RESULTS

**FIGURE 1:**
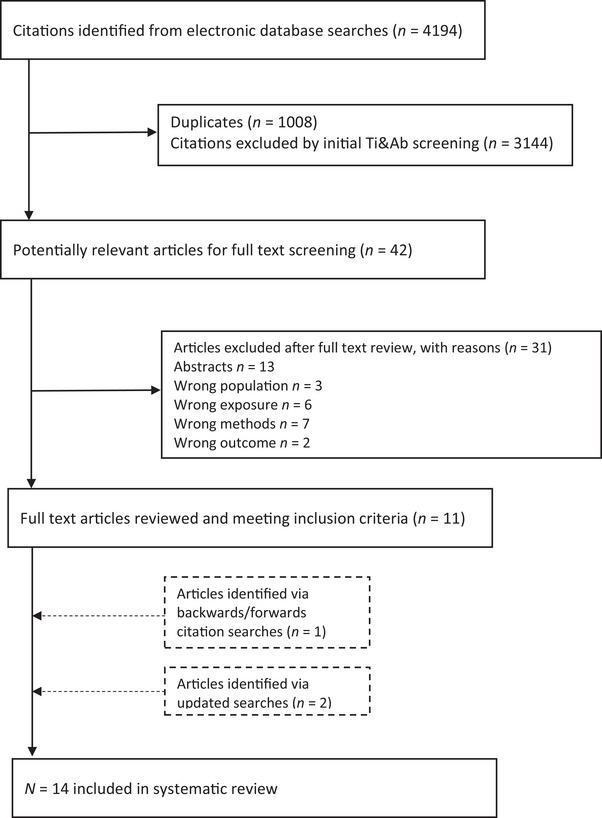
Flow chart of the study selection process.

The electronic searches yielded 4194 citations. After removing duplicates, 3186 titles and abstracts were screened identifying 42 articles for full‐text review. Eleven studies met the inclusion criteria, one study was identified through citation chasing and two additional studies were identified through the updated searches. Fourteen studies were included in total. The selection process is summarized in Figure 1.

### 1 Study characteristics

A summary of the characteristics of included studies is shown in Table 1. Most of the studies used data from the US (*n* = 9), with the remaining studies using data from Japan (*n* = 2), South Korea (*n* = 1), Germany (*n* = 1), and Wales (*n* = 1). Six studies used a cohort design,^17^, ^24^, ^25^, ^26^, ^27^, ^28^ six were case‐control studies,^16^, ^29^, ^30^, ^31^, ^32^, ^33^ one reported a self‐controlled cohort design,^15^ and one study was designed as multiple emulated clinical trials.^34^ Administrative claims data were the most used data type (*n* = 7), followed by EHRs (*n* = 6), while one study used both EHRs and claims data. Sample size varied across studies ranging from 7500 to 117 million (total 139,096,622 participants) with 1,098,157 cases of dementia identified in total (note: information not provided in all studies).

**TABLE 1:** Characteristics of 14 included studies reporting data‐driven associations of medications with dementia risk.

Study, country	Study design	Purpose of data‐driven approach	Type of dataset used	Sample size	(ML/AI) Methods used	Predictors (features) used	Time window	Main findings in relation to prescribed medication
Hu (2023), USA	Matched case‐control	Examine association between medications and dementia incidence	ACD	Cases = 835 Controls = 101,084	PS‐matched survival analysis	200 most common medications taken by AD group	2012–2021	18 medications associated with ↓ risk (eg, antibiotics, anti‐inflammatories) 10 medications associated with ↑ risk (eg, anti‐dementia drugs, antidepressants)
Kern (2019), USA	Self‐controlled cohort	Examine association between medications and dementia incidence	ACD (4 databases)	117,015,066	Incidence rate ratio, meta‐analysis	2181 medications	Treatment duration 3–12 months	17 medications associated with ≥50% ↓ risk of dementia in ≥2 databases: catecholamine modulators, anticoagulants, anticonvulsants, antibiotics/antivirals, and a miscellaneous group (acamprosate, quinidine, palonosetron, pegfilgrastim)
Nakaoku (2021), Japan	Retrospective cohort	Identify dementia, AD, VaD predictors	ACD	226,738	Logistic regression	11 medication codes, demographics, 22 medical diagnoses	FU: 5 years	All‐cause and AD: antidepressant, antipsychotic, hypnotic, antithrombotic use ↑ risk; All‐cause and AD: antihypertensive and dyslipidemia drugs ↓ risk VaD: Antidepressant, antipsychotic use ↑ risk; Dyslipidemia drugs use ↓ risk
Wilkinson (2021), Wales	Retrospective cohort	Examine association between medications and AD and dementia incidence	EHR	551,344	Survival analysis	744 medications, demographics, SES, smoking status	Median FU time for those who developed dementia = 17.0 years, for those who did not receive a dementia diagnosis = 9.7 years	217 medications ↑ risk (eg, cardiovascular diseases, diabetes, gastroesophageal reflux disease, laxatives, hypnotics, anxiolytics, antiepileptics, antimalarials) 4 vaccines ↓ risk (hepatitis A, typhoid, hepatitis A and typhoid combined, diphtheria)
Fukunishi (2020), Japan	Retrospective cohort	Prediction of AD	ACD	48,123	Sparse Logistic regression	107 medications, demographics, 502 medical diagnoses	FU: 31 months	NSAIDs only medication feature with high appearance frequency in both prediction models
Mayburd (2019), USA	Retrospective cohort	Discover possible combinations of drugs associated with reduced dementia risk	EHR	∼50,000	Built linear model to fit signals found in EHR data to clinical trial performance following review of compounds in >300 studies	1900 medications and supplements into 53 mechanism‐based groups, demographics, education, comorbidities	FU: 5 years	Strongest negative correlations with diagnosed dementia and/or dementia progression rate were identified for herbals, Zn, Se, Mg, biotin, vitamin A, lutein and chromium picolinate. In context of EMR, the supplements were often reported in combinations
Miled (2020), USA	Matched case‐control	Prediction of dementia onset	EHR	Cases = 2159 Controls = 11,558	RF	235 features inc. 100 medication groups, demographics, institution, 19 diagnoses, 110 medical notes report types	1‐ and 3‐year prediction prior to diagnosis	Antidepressants, diuretics, antihyperlipidemics, antihypertensives, opioid‐analgesics as top features in predictive models
Nori (2019), USA	Nested case‐control	Prediction of dementia incidence	ACD	Cases = 44,945 Controls = 760,646	Lasso, Logistic regression	Top 50 predictors inc. medication codes, demographics, diagnosis and procedure codes	4–5 years prediction prior to diagnosis	18 medications among the top 50 predictors in the model, with the top 5 codes being psychoactive drugs and meds related to vascular disease and diabetes ↑ risk
Park (2020), South Korea	Retrospective cohort	Predict AD incidence	ACD	40,736	Logistic regression, SVM, RF	4894 features inc. medications, socio‐demographics, lab tests, diagnoses, history of personal and family illness	0–4 years prior to diagnosis	4 medications among top 10 features from LR: Zotepine (antipsychotic), Eperisone hydrochloride (antispasmodic) ↑ risk nicametate citrate (vasodilator), tolfenamic acid (analgesic) ↓ risk
Reinke (2022), Germany	Retrospective cohort	Predict risk of dementia incidence	ACD	117,895	Logistic regression, GBM, RF	324 features including 212 medication codes, demographics, 87 procedure codes, 23 diagnoses	FU: 10 years	GBM best calibration model. 10 medications in LR model and 11 in GBM model among top 20 features including: Antipsychotics, anti‐dementia drugs, antidepressants, psychostimulants, urologicals, insulins and analogues ↑ risk Cough suppressants, macrolides, lipid modifying agents, angiotensin receptor blockers ↓ risk
Xu (2020), USA	Matched case‐control	Prediction of AD	EHR	7587	Logistic regression, Lasso, RF, XGBoost	171 medication classes, demographics, 32 diagnoses	0–3 years prior to diagnosis	13 drug groups as top features: anti‐dementia drugs, antipsychotics, antiepileptics, angiotensin II antagonists, adrenergics, anti‐inflammatory agents, antivirals, antidepressants
Xu (2022), USA	Matched case‐control	Validate associations of top candidate drugs (identified via a genetics and multiomics network‐based system) in relation to AD risk	EHR	3,180,111	Network‐based system, then PS‐matched survival analysis	118 prioritized medications for AD treatment reduced to 4 for survival analysis	2011–2021	4 medications associated with ↓ risk: gemfibrozil (reduction of triglycerides), ibuprofen (anti‐inflammatory), ceftriaxone (antibiotic), cholecalciferol (vitamin D3)
Zang (2023), USA	Emulated clinical trials	Identify non‐AD drug candidates that can be repurposed for treating AD	EHR, ACD	498,888	PS calculation models including regularized logistic regression, long short‐term memory network, gradient boosted decision trees, and multi‐layer perceptrons	312 medications (OneFlorida = 66, MarketScan = 246), demographics, diagnosis codes, time from MCI date to drug trial date	FU: 5 years	5 drugs showed consistent beneficial ^a^ effects on both datasets: pantoprazole, omeprazole (gastroesophageal reflux disease), gabapentin (antiepileptic), atorvastatin (high cholesterol), fluticasone (nasal symptoms, asthma)
Zhou (2020), USA	Case‐control	Corroborate top candidate drugs (identified via a drug‐target interactions prediction system) in relation to AD and dementia risk	EHR	∼17,000,000	Network‐based drug‐target interaction prediction system, then Logistic regression	1430 drugs, 4251 side effects, 1059 diseases, and 17,860 genes, then top 30‐ranked repositioned drug candidates	not specified	Drugs to treat diabetes, hypertension, hyperlipidemia, obesity, cancer, myasthenia gravis among the top‐ranked drugs associated with ↓ risk Some statins associated with ↑ risk

Abbreviations: ↑, increased risk; ↓, reduced risk; ACD, administrative claims/health data; AD, Alzheimer's disease; AI, artificial intelligence; EHR, electronic health records data; EMR, electronic medical record data; FU, follow‐up; GBM, gradient boosting machines; LR, logistic regression; MCI, mild cognitive impairment; ML, machine learning; NSAIDs, non‐steroidal anti‐inflammatory drugs; PS, propensity score; RF, random forest; SES, socioeconomic status; SVM, support vector machine; VaD, vascular dementia.

^a^ Plus additional drugs associated with reduced risk identified in one of the two databases; see Table S3.

### 2 Study methods and focus

All included studies used data‐driven methods to examine associations between prescribed medications and dementia. However, seven of the studies^15^, ^24^, ^26^, ^30^, ^31^, ^33^, ^34^ focused specifically on medications, whereas the rest^16^, ^17^, ^25^, ^27^, ^28^, ^29^, ^32^ applied ML methods to predict AD or all‐cause dementia risk using a range of available features including medications in their models. Of the seven former studies, three were medication‐wide studies using all available medications in the database.^15^, ^24^, ^30^ One study^26^ produced hazard ratios for dementia risk validated by clinical trials, studies, and animal experiments for a given agent which were then ranked based on specified group metrics and the top‐ranking agents were traced as combinations of agents in patient profiles. One study identified drug candidates to be repurposed for AD treatments by emulating 430,000 trials ^34^ and two studies corroborated top candidate drugs identified via a drug‐target interactions prediction system,^33^ and a genetics and multiomics network‐based system,^31^ respectively.

The studies using ML techniques (*n* = 7) considered a variety of predictors or sets of features including the following: demographics, education, medical diagnoses and International Classification of Diseases (ICD) codes, procedures, medications, medical notes, laboratory tests, history of personal and family illness, health service utilization, side effects, and genes. Logistic regression was the most common modeling technique (*n* = 6), followed by random forest (*n* = 4), gradient boosting trees (*n* = 2), lasso (*n* = 2), XGBoost (*n* = 1), support vector machine (*n* = 1), sparse logistic regression (*n* = 1), regularized logistic regression (*n* = 1), and long short‐term memory network, multilayer perceptrons, and a network‐based drug‐target interaction prediction system (*n* = 1 each). Five studies compared the ML algorithms with other methods.^17^, ^25^, ^28^, ^32^, ^34^ Random forest outperformed logistic regression in one study,^28^ regularized logistic regression outperformed deep learning‐based methods in another study,^34^ while logistic regression showed comparable performance to random forest and gradient boost machine/XGBoost in two studies.^17^, ^32^ The most commonly reported performance measure was the area under the receiver operating characteristic curve (AUC) with values across studies ranging from 0.64 to 0.94.

### 3 Medication assessment

Prescription information within studies was most often mapped to ingredients or drug classes, although it was not always clear which classification system was used to derive those. The Anatomical Therapeutic Chemical Classification,^17^, ^27^, ^31^, ^33^, ^34^ British National Formulary,^24^ the Generic Product Identifier,^29^, ^34^ and the Hierarchical Ingredient Code list^16^ were among those reported. Eight studies included all medications identified within the database used under specified thresholds (eg, prescribed to ≥500 or ≥1000 participants), one study preselected medication groups based on associations reported in the literature,^27^ two studies selected the top‐ranked drug candidates following modeling of phenotypic and genetic relationships,^31^, ^33^ whereas the rationale for medication inclusion was not clear in three studies.^16^, ^28^, ^32^ “Exposed” or prevalent users were generally defined as those who had been prescribed any of the medications at least once. One study considered time on treatment^15^ and another study investigated the presence of multiple ingredients in patient profiles.^26^ Although there are differences in terms of study categorization and reporting of medications, we estimated that the included studies examined more than 200 pharmacological subgroups including more than 2000 ingredients. Associations between medication and dementia risk were considered based on a *p*‐value of <0.05; individual studies applied additional specifications as described in the original papers.

### 4 Dementia assessment

Dementia was identified by ICD‐9 or ICD‐10 codes in EHRs and claims data or in combination with prescriptions and pharmacy claims for donepezil, galantamine, rivastigmine, or memantine. Details of dementia diagnosis were unclear in two studies.^26^, ^33^ AD was an outcome in the majority of studies (*n* = 9), while seven studies reported all‐cause dementia, and one study also included results for vascular dementia.^27^

### 5 Study quality assessment

Study quality was assessed either by PROBAST^22^ (seven studies) or JBI (six studies) checklists^23^ depending on the purpose of the data‐driven approach (Table S2). The tools were not applicable to the study by Mayburd et al.^26^ When PROBAST was used, risk of bias was judged as “low” in 71% of the studies for the participants domain, 86% for the predictors and outcomes domains, and 71% for the analysis domain. Low risk of bias across all four domains was observed in five^16^, ^17^, ^25^, ^28^, ^32^ out of the seven studies. One study^29^ received a “high” overall risk of bias and for one study the overall risk of bias was estimated as “unclear.” For all six studies ^15^, ^24^, ^27^, ^30^, ^31^, ^33^ assessed by JBI checklists, risk of bias was “low” regarding the questions covering participant selection, while five of the studies scored a low risk of bias for outcome assessment.^15^, ^24^, ^27^, ^30^, ^31^ However, risk of bias was estimated as potentially “high” for four^24^, ^27^, ^30^, ^31^ of the studies for questions covering exposure assessment, and for two studies as “unclear” for questions assessing loss to follow‐up. Medication‐wide studies used Bonferroni correction,^24^ Benjamini–Hochberg method^24^, ^30^ or a combination of other criteria to identify medications that passed their defined threshold and were associated with dementia.^5^, ^30^ ML studies often used ensemble methods to improve overall accuracy of prediction models including random forest,^17^, ^28^, ^29^, ^32^ gradient boosting,^17^, ^32^ and long short‐term memory network.^34^

### 6 Medications associated with reduced dementia risk

Ten studies^15^, ^17^, ^24^, ^26^, ^27^, ^28^, ^30^, ^31^, ^33^, ^34^ reported associations between a range of medications and reduced risk of all‐cause dementia (*n* = 6), AD (*n* = 7), or vascular dementia (*n* = 1). Three were medication‐wide association studies.^15^, ^24^, ^30^ Using different study designs and threshold criteria for medication inclusion and risk reduction in their analyses, the studies identified associations with 17 medications (≥50% reduction: catecholamine modulators, anticoagulants, anticonvulsants, antibiotics/antivirals, other group),^15^ and four vaccines (hepatitis A, typhoid, hepatitis A and typhoid combined, diphtheria),^24^ respectively. Associations with the four vaccines were reported for both all‐cause dementia and AD with hazard ratios ranging from 0.68 to 0.92.^24^ Fifteen additional medications were associated with reduced AD risk in the third medication‐wide study,^30^ falling mainly under the categories of antibiotics and anti‐inflammatory drugs. When the same study examined specifically repurposed drug candidates from clinical trials that were also available in their drug list data, three additional medications were found to be associated with reduced risk of AD: valacyclovir (antiviral), montelukast (inflammation), and losartan (antihypertensive) (hazard ratio [HR] range 0.56 to 0.73).^30^

Three additional studies focused on drug repurposing specifically for AD.^31^, ^33^, ^34^ One of the studies developed a network‐based drug‐target interactions prediction system by modeling thousands of phenotypic and genetic relationships, followed by clinical corroboration of the top repositioned drug candidates using EHR population‐level data.^33^ Similarly, Xu et al.^31^ used a genetics and multiomics network‐based system to identify top candidate drugs for AD and validated potential associations using EHR data. Medications to treat diabetes, hypertension, hyperlipidemia, obesity, and treatments for myasthenia gravis and cancer (breast, prostate) were among the top‐ranked drugs associated with lower odds of AD and dementia diagnosis in the former study.^33^ Four different medications were identified in the second network‐based study^31^ to be associated with decreased AD risk, namely gemfibrozil (reduction of triglycerides), ibuprofen (anti‐inflammatory), ceftriaxone (antibiotic), and cholecalciferol (vitamin D3) (odds ratio [OR] range of 0.76 to 0.86). The third study^34^ emulated thousands of drug trials (a process that imitates targeted randomized controlled trials [RCTs]) using two large‐scale databases and identified several approved drugs associated with reduced risk of mild cognitive impairment to AD progression. Five of those drugs showed consistent beneficial effects on both datasets ranging from 6% to 26% risk reduction. Original indications of those five drugs were for gastroesophageal reflux disease, epilepsy, asthma, and high cholesterol.

Two studies reported on data‐driven ML models to predict AD risk.^17^, ^28^ The study by Park et al. used administrative health data from Korea and implemented three ML algorithms. However, only the top 10 features from logistic regression for 0‐year prediction were reported, identifying two medications associated with lower AD incidence: a vasodilator (OR = 0.74) and a pain killer (OR = 0.77).^28^ The second study also applied three ML models and identified several medications as important predictors in both logistic regression and gradient boosting machine models. Medications associated with reduced risk were the following: cough suppressants, antibacterials, lipid modifying agents, and angiotensin II receptor blockers (ORs ranging from 0.86 to 0.90).^17^

In a study using claims data covering Japanese citizens, use of antihypertensive and dyslipidemia medications was associated with lower risk of all‐cause dementia, AD, and vascular dementia (dyslipidemia medications only) diagnosis. ORs ranged from 0.73 to 0.96.^27^ This study included only 11 medication codes among other predictors of dementia diagnosis.

Using data from three US databases, Mayburd and colleagues^26^ generated mechanism‐based groups based on more than 1900 approved drugs and supplements and aligned those with data presented in 300 clinical trials and animal studies. They then built a model to fit the signals from the EHRs to clinical trial performance. The larger groups showing promise included cerebrovascular modulators (antihypertensives, vasodilators, PDE5 inhibitors, antiplatelet, antimigraine), immunomodulators (antihistamine, non‐steroidal anti‐inflammatory drugs [NSAIDs], antivirals, antigout, and anti‐arthritis) and metabolic stimulators, coenzymes, antioxidants, and vitamins. The higher‐ranked pharmacological combinations were used to produce protective complexity scores within patient profiles. Their analysis showed that the higher the number of top‐ranking active agents present in a patient's profile the lower was the fraction of dementia. The strongest negative correlations with dementia or progression of cognitive decline were identified for combinations of herbals, zinc, selenium, magnesium, biotin, vitamin A, lutein, and chromium picolinate.

Overall, medication classes associated with reduced risk of AD in multiple studies included antibiotics (*n* = 5 studies), antihypertensives (*n* = 6), lipid‐lowering drugs (*n* = 5), anti‐inflammatories (*n* = 4), and vaccines/antivirals (*n* = 3). Most studies focused on AD though similar results were seen for all‐cause dementia, perhaps because of diagnostic overlap given the bulk of dementia cases are caused by AD. There was limited overlap of specific agents indicating a significant association with reduced dementia risk across studies/datasets, except the following nine drugs which appeared in two studies each: amoxicillin, azithromycin, doxycycline, fluticasone, ibuprofen, losartan, methylprednisolone, mirtazapine, and prednisone. In a sub‐analysis using the Wilkinson paper^24^ as the base for comparisons due to its size, medication coverage and duration of follow‐up, four specific medications were identified as related to reduced risk of all‐cause dementia in both the Wilkinson paper (in the 5‐year sensitivity analysis, data not shown) and additional studies: atorvastatin, cholecalciferol (vitamin D3), omeprazole, and gabapentin. See Table S3 for medications associated with reduced risk, the papers in which they were identified, their World Health Organization (WHO) anatomical therapeutic chemical classification and indication.

### 7 Medications associated with increased dementia risk

Ten studies reported medication associations with increased risk of all‐cause dementia (*n* = 6), AD (*n* = 7), or vascular dementia (*n* = 1) (Table 1). In one of the medication‐wide studies,^24^ 217 out of the 744 medications were associated with increased risk including medications expected a priori with indications for cardiovascular disease, diabetes, depression, neurodegenerative diseases, and symptoms or complications of dementia. Additional medications found to be associated with increased risk were for conditions such as dyspepsia and gastroesophageal reflux disease (HR range 1.14 to 1.63), drugs used in nausea and vertigo (HR range 1.20 to 2.88), laxatives (HR range 1.39 to 2.27), proton pump inhibitors, hypnotics and anxiolytics (HR range 1.27 to 3.37), analgesics (HR range 1.22 to 1.89), anticonvulsants (HR range 1.53 to 4.99), drugs used in substance abuse, and drugs for genitourinary disorders (HR range 1.38 to 3.44). Of note, some of these medications have also been found to be associated with reduced risk (see previous section). However, in a sensitivity analysis including drugs first prescribed 10 or more years before diagnosis, it was primarily antidepressants and antipsychotics that were still associated with higher dementia incidence.^24^ In the medication‐wide study by Hu et al.^30^ medications associated with increased AD risk included several used for depression/anxiety (sertraline, escitalopram, trazodone, mirtazapine) and antipsychotics (quetiapine). When associations were examined only for drugs from clinical trials for treating AD, again medications indicated for depression/anxiety, insomnia, seizures, and Parkinson's showed increased risk (HR range 3.11 to 4.03).^30^

In the study by Zhou et al.^33^ where the top repositioned drug candidates were clinically corroborated using EHR population‐level data, statins (fluvastatin, pravastatin), sulfamethoxazole (antibacterial), and pioglitazone (for diabetes), were associated with increased AD risk (adjusted OR range 1.14 to 1.32). Top‐ranked drugs associated with all‐cause dementia in addition to those for AD risk were as follows: neostigmine (used in the treatment of myasthenia gravis), disulfiram (alcohol dependence), gemfibrozil (hyperlipidemia), anastrozole (breast cancer), prednisone (inflammatory conditions), and etoricoxib (anti‐inflammatory analgesic). In the study using claims data covering Japanese citizens,^27^ antidepressants, antipsychotics, hypnotics, and antithrombotic medications predicted all‐cause dementia and AD. Vascular dementia predictors included antidepressants and antipsychotics (OR range 1.06 to 2.25).^27^

Six studies reported on ML models to predict AD/dementia risk in individuals aged 70 years and older. In a study using regularly collected claim data and aiming to predict AD risk in people over 75 years old,^25^ use of NSAIDs was the only medication predictor with high appearance frequency among the 13 features selected in the model with best performance. Using among others a prescriptions‐focused model, Miled et al. showed that the model could predict dementia 1 year and 3 years prior to diagnosis with an accuracy of 70% and 65% respectively; antidepressants, diuretics, antihyperlipidemics, and antihypertensives were among the top predictive features.^29^ Aiming for a 4‐ to 5‐year prediction prior to dementia diagnosis, the final model by Nori et al. included 18 medications among the top predictors; antidepressants were among the top five medications followed by anticholinergics (for overactive bladder), pain killers, and drugs to treat vascular disease and diabetes.^16^ In a smaller EHR dataset from the US, Xu et al.^32^ developed several ML models for 0‐ to 3‐year prediction windows of AD risk, all achieving an AUC above 73%. Top featured medications verified by XGBoost included anti‐dementia drugs (eg, memantine), antipsychotics, antiepileptics, angiotensin II antagonists, adrenergics, anti‐inflammatory agents, antivirals, and antidepressants.^32^ Two medications, an antipsychotic (zotepine) and an antispasmodic (eperisone), were identified among the top selected features from logistic regression in Park et al.’s^28^ model. Despite developing a 0‐ to 4‐year AD risk prediction model, important features were only reported for the 0‐year incidence.^28^ Finally, Reinke et al.^17^ developed models to predict dementia incidence including more than 300 features from German claims data. Nearly half of the top features were prescribed medications associated with increased risk including antipsychotics, antidepressants, psychostimulants, urologicals, and insulin preparations.^17^

Overall, use of antidepressants, antipsychotics, and medications for cardiovascular disease and diabetes were among the top predictors in several ML models developed for the early identification of dementia using EHR or claims data.^16^, ^17^, ^28^, ^29^, ^32^ A similar pattern of associations was observed in the non‐ML studies. There was minimal overlap of specific agents indicating a significant association with increased dementia risk across studies/datasets, with 13 out of more than 200 drugs appearing in two or three studies: citalopram, escitalopram, levetiracetam, metformin, mirtazapine, oxybutynin, quetiapine, sertraline, simvastatin, sulfamethoxazole, tramadol, trazodone, and venlafaxine. The same medications were identified in the sub‐analysis using the Wilkinson paper as the base for comparisons in the overall dementia risk and partly in the 5‐year sensitivity analysis (data not shown), with the addition of two agents: duloxetine and clopidogrel. See Table S4 for medications associated with increased risk, the papers in which they were identified, and their WHO anatomical therapeutic chemical classification and indication.

## 3 . DISCUSSION

Focusing on studies using a data‐driven approach to investigate associations between prescribed medications and dementia risk, we identified 14 studies including administrative or medical records data for more than 130 million individuals. Such studies are at risk of producing false positive associations, and though all studies allowed for this in their analysis, this systematic review provides an important further check by looking for consistency of signal across different papers, datasets, and methodologies. Overall, we found a lack of consistency between studies in identifying individual drugs which modify risk of all‐cause dementia or AD. However, some drug classes with biological plausibility were identified including antimicrobials, vaccines, anti‐inflammatories, and antihypertensives for reduced risk; and antipsychotics, antihypertensives, drugs for diabetes, and antidepressants for increased risk. Some drugs featured in lists of those that were associated with both increased and decreased risk. There are a number of possible explanations for this. The first is that the data used to perform the analyses were not appropriate to identify robust associations or questions of causality. These are naturalistic datasets which have been accumulated for clinical, not research, purposes and they will therefore be suboptimal in a number of ways. All observational cohort data are subject to confounding variables which may mediate or attenuate any effect which may mask any link between exposure and outcome. These datasets may have substantial amounts of missing or incorrectly entered data. For the results that are obtained it is impossible to assess the direction of causality. For example, antidepressants prescribed in early stages of dementia presenting with altered mood would be associated with an increased likelihood of dementia diagnosis, though it is dementia which increases the risk of being prescribed antidepressants (reverse causation).

Grouping drugs into apparently similar classes may lead to conflicting results due to differing effects of different class members. For example, autophagy upregulation has been suggested as a potential disease‐modifying pathway. Many commonly prescribed antihypertensives (eg, some calcium channel antagonists) upregulate autophagy in animal models and can rescue neurodegenerative phenotypes.^35^ However, other antihypertensives do not cross the blood‐brain barrier or do not upregulate autophagy and therefore analysis of antihypertensives as a class may mask beneficial effects of particular agents. Analyzing the results in other ways (for example specific biological pathways or particular receptor agonism) might yield different results. Despite these challenges, the numbers of people involved would seem to make a type II error unlikely. An alternative possibility is that there are no routinely prescribed individual medicines which alter the risk of dementia, though this also seems unlikely given the number of drugs investigated and their myriad pleiotropic mechanisms of action.

Despite the lack of consistency for individual drugs, there are some themes that emerge for drug classes and are consistent with previously published literature and biological plausibility. The association between antibiotics, antivirals, and vaccines and decreased risk of dementia is intriguing. Viral and bacterial infectious causes of common dementias have been proposed, supported by epidemiological data linking infection to dementia risk, antiviral drugs have been identified as some of the most promising repurposed drugs for dementia, and there is increasing interest in vaccination as being generally protective.^36^, ^37^, ^38^ Our findings support these hypotheses and lend further weight to these agents as being potentially disease‐modifying or preventative for dementia. Antihypertensives feature as drugs which may decrease risk in several studies, despite being prescribed for hypertension which is itself a risk factor for dementia. This may be due to specific effects of some antihypertensives^39^ or as a class effect by improving vascular health. Treating hypertension in midlife is something with pleiotropic positive benefits and our findings support the identification and treatment of hypertension and hyperlipidemia in midlife. The next most common group of medications associated with decreased risk were anti‐inflammatories. This fits with the increased interest in inflammation being a significant pathogenic pathway, partly stimulated by genetic data supporting this hypothesis.^40^ Agents addressing anti‐inflammatory targets are among the largest categories in the drug development pipeline for AD.^41^ Although some early clinical trials of these agents have yielded null results,^42^ using the right agent at the right time point in disease progression, perhaps prior to manifestation of cognitive decline, may be crucial and this is not covered in the studies included in the review.

In terms of increased risk, antipsychotic medication appeared strongly. Though, as with antidepressants, reverse causation cannot be ruled out and indeed is likely to explain at least some of this association, the finding is consistent with previous literature and serves as a useful reminder of the need for caution with these agents, particularly in populations at risk of, or already diagnosed with, dementia.^43^ Understanding the mechanism of this potential effect and whether it is a true class effect or whether risk varies by specific drug would be useful to help inform clinical practice. Antidepressants and other drugs targeting the nervous system, and to a lesser extent drugs prescribed to manage blood glucose levels, were among the groups associated with both reduced and increased dementia risk in some of the reviewed studies. Although these observations may reflect methodological differences such as thresholds for medication inclusion and time windows of relevant analyses, it is worth noting that those categories of medications were among the few that appeared to be significantly associated with increased dementia risk in two or more datasets. Besides being among widely prescribed medications, several agents in these drug classes are proposed repurposed drugs in the current AD pipeline for prodromal or mild to moderate dementia, for example, metformin and escitalopram.^41^

Limitations of the reviewed studies need to be acknowledged. Data on drug administration were not available and prescribed medication was considered as “exposure” although this does not guarantee actual intake. Similarly, individuals may have used a drug without making a prescription claim, and therefore exposure misclassification is possible. Dose‐response relationships or individual pharmacogenomic responses were not examined within the reviewed studies. The role of single versus multiple medications in dementia risk would also be informative given the increased rates of multimorbidity as populations age and adverse effects are potentially associated with polypharmacy. Furthermore, bias is possible in pharmacoepidemiologic studies due to the unavailability of confounding factors such as education level, socioeconomic status, and genotyping (eg, apolipoprotein E [*APOE*]) or biomarker information (eg, p‐tau‐217). Several included studies used approaches to reduce the impact of confounding variables including adjustments for age, gender, smoking status,^24^ comorbidities,^27^, ^31^ using a self‐controlled study design^15^ or propensity matching^30^, ^34^ for analyses. ML studies used numerous variables as model predictors including basic sociodemographic variables, medical diagnoses, laboratory tests, and genome‐wide association studies (GWAS) and multi‐omics findings to identify dementia risk predictors or drug repurposing candidates.

Reverse causality is also plausible and has been discussed in many of the included studies to interpret observed associations. Although the clustering of medications around certain indications may aid the identification of common symptoms preceding dementia diagnosis, they may dilute true associations with dementia risk. Study designs with appropriate lag time—removing years of observation time directly before the outcome—might partly address this issue and be rare in the current synthesis. Only one study conducted a sensitivity analysis excluding medications first prescribed 5 and 10 years before diagnosis and identified very few drugs still being associated with higher dementia incidence, namely antidepressants and antipsychotics.^24^ There is also potential inaccuracy in terms of the outcome, as dementia may be under‐ or misdiagnosed. Several studies used a combination of diagnosis codes and anti‐dementia drug prescriptions and/or consecutive claims to identify dementia cases, and future studies could complement their dementia identification algorithms with information within clinical notes through natural language processing.^44^ Various medications featured in the studies applying ML approaches to develop dementia risk prediction models. Although the models generally showed acceptable discriminatory power, external validation was not performed and is required before considering their potential as supporting tools for risk prediction. Finally, although we conducted comprehensive and updated searches, the interest and application of data‐driven approaches utilizing medical and insurance claims data to predict disease risk including dementia and AD is growing rapidly and we may have missed recent publications.

The work presented here is the largest systematic review of studies using a data‐driven approach to investigate associations between medications currently in use and risk of developing dementia. Though the results are not immediately clear‐cut for individual drugs, some expected and some unexpected patterns have emerged. Understanding whether drugs in current use could be repurposed for use in dementia is an urgent priority and will become more important with the emergence of platform trials in the field. Future studies augmenting data‐driven approaches using omics and drug target Mendelian randomization for drug discovery and causality assessment can contribute to this direction. The work presented here can be used to help prioritize drug repurposing candidates and provides a basis for further exploration of these datasets and potential pathogenic pathways.

## AUTHOR CONTRIBUTIONS

This article is the product of the DEMON Network Drug Discovery and Trial Optimization Working Group. Benjamin R. Underwood and David J. Llewellyn contributed to the original study proposal. Ilianna Lourida drafted the original protocol with revisions by Benjamin R. Underwood and David J. Llewellyn. Ilianna Lourida led the screening, data extraction, and quality assessment process. Ilianna Lourida, Jessica Gong, Stefano Tamburin, Eugene Yee Hing Tang, Emad Sidhom, Xin You Tai, Matthew J. Betts, Janice M. Ranson, Margarita Zachariou, Olajide E. Olaleye, and Saswati Das independently screened papers and Ilianna Lourida, Neil P. Oxtoby, and Shanquan Chen assessed the methodological quality. Benjamin R. Underwood, Ilianna Lourida, and David J. Llewellyn synthesized the data, and Ilianna Lourida and Benjamin R. Underwood wrote the draft report and are joint first authors. All authors critically reviewed the manuscript for important intellectual content, approved the final submitted version, and accepted responsibility to submit it for publication.

## CONFLICT OF INTEREST STATEMENT

BRU has been part of advisory boards for Lilly and TauRx. He is part of the UK consortium designing the MASTODON adaptive platform trial in AD. NPO is a paid consultant for Queen Square Analytics Ltd (UK) on projects involving AD and clinical trials. All other authors declare no competing interests. Author disclosures are available in the supporting information.

## CONSENT STATEMENT

It was not necessary to confirm consent of individuals for this work as it is a secondary analysis of already published data.

## Supporting information



Supporting Information

Supporting Information
