# Supplementary material for: Data‐driven discovery of associations between prescribed drugs and dementia risk: A systematic review
Source: Alzheimers Dement (N Y). 2025 Jan 21;11(1):e70037. doi: 10.1002/trc2.70037 (PMC11747987; doi:10.1002/trc2.70037)
Supplement: Supplementary file 1 — Supporting Information [file TRC2-11-e70037-s002.pdf]

Supplementary Table 1. Search strategy via Ovid

Medline

Ovid MEDLINE(R) and Epub Ahead of Print, In-Process, In-Data-Review & Other Non-Indexed Citations and Daily <1946 to August 04, 2022>

1 exp Pharmaceutical Preparations/
2 exp Prescriptions/
3 (drug* or treatment* or medicat* or prescri*).tw,kf.
4 exp Dementia/
5 exp Alzheimer Disease/
6 (dement* or Alzheimer*).tw,kf.
7 exp Medical Records Systems, Computerized/ or exp Electronic Health Records/ or exp Health Records, Personal/ or exp Routinely Collected Health Data/ or exp Patient Generated Health Data/ or Insurance Claim Reporting/
8 (electronic patient record* or electronic medical record* or electronic health record* or computerized patient record* or computerized medical record* or computerized health record* or ambulatory medical record* or medical record* or health record* or hospital record* or personal health record* or personal medical record* or personal electronic health record* or electronic personal health record* or personal health information or medical records system* or computerized medical records system* or patient generated health data or administrative data or administrative claim data or electronic health data* or administrative health data* or claims data*).tw,kf.
9 1 or 2 or 3
10 4 or 5 or 6
11 7 or 8
12 9 and 10 and 11

EMBASE

Embase <1974 to 2022 August 04>

1 exp Pharmaceutical Preparations/
2 exp Prescriptions/
3 (drug* or treatment* or medicat* or prescri*).tw,kf.
4 exp Dementia/
5 exp Alzheimer Disease/
6 (dement* or Alzheimer*).tw,kf.
7 exp Medical Records Systems, Computerized/ or exp Electronic Health Records/ or exp Health Records, Personal/ or exp Routinely Collected Health Data/ or exp Patient Generated Health Data/ or Insurance Claim Reporting/
8 (electronic patient record* or electronic medical record* or electronic health record* or computerized patient record* or computerized medical record* or computerized health record* or ambulatory medical record* or medical record* or health record* or hospital record* or personal health record* or personal medical record* or personal electronic health record* or electronic personal health record* or personal health information or medical records system* or computerized medical records system* or patient generated health data or administrative data or administrative claim data or electronic health data* or administrative health data* or claims data*).tw,kf.
9 1 or 2 or 3
10 4 or 5 or 6
11 7 or 8
12 9 and 10 and 11

PsycINFO

APA PsycInfo <1806 to July Week 4 2022>

1 exp Drugs/ or exp Prescription Drugs/
2 (drug* or treatment* or medicat* or prescri*).tw.
3 exp Dementia/

4	exp Alzheimer's Disease/
5	(dement* or Alzheimer*).tw.
6	exp Electronic Health Records/ or exp Medical Records/ or exp Health Information/
7	Medical Records Systems, Computerized.mp.
8	Health Records, Personal.mp.
9	Patient Generated Health Data.mp.
10	Routinely Collected Health Data.mp.
11	Insurance Claim Reporting.mp.
12	(Electronic patient record* or Electronic medical record* or Electronic health record* or Electronic health data or Computerized patient record* or Computerized medical record* or Computerized Medical Records System* or Ambulatory medical record* or Medical record* or Health record* or Hospital record* or Personal health record* or Personal medical Record* or Personal electronic health record* or Electronic personal health record* or Personal health information or Medical Records System* or Administrative data or Administrative claim data or Administrative health data).tw.
13	1 or 2
14	3 or 4 or 5
15	6 or 7 or 8 or 9 or 10 or 11 or 12
16	13 and 14 and 15

Supplementary Table 2. Risk of bias assessment using PROBAST and JBI

Study	Participants	Predictors	Outcome	Analysis
**PROBAST**				
Ben Miled (2020)	?	+	+	-
Fukunishi (2020)	+	+	+	+
Mayburd (2019)	n/a	n/a	n/a	n/a
Nori (2019)	+	+	+	+
Park (2020)	+	+	+	+
Reinke (2022)	+	+	+	+
Xu (2020)	+	+	+	+
Zang (2022)	?	?	?	?
**JBI**				
Hu (2023)	+	-	+	?
Kern (2019)	+	+	+	+
Nakaoku (2021)	+	-	+	?
Xu (2022)	+	-	+	?
Wilkinson (2021)	+	-	+	?
Zhou (2020)	+	+	?	?

+ indicates low RoB; – indicates high RoB; ? indicates unclear RoB.
Risk of bias assessment using the JBI was fitted into the PROBAST domains for the purpose of summarising the assessments in a single table.

Supplementary Table 3. List of medications associated with reduced AD or dementia risk, ATC* level and indication					
**Medication name/class**	**Study (N sample size)****	**Finding or estimate of association (95% confidence interval)**	**ATC-Level 1**	**ATC-Level 3**	**Indication**
abiraterone	Zhou	D: adjusted OR=0.81 (0.73-0.91) AD: adjusted OR=0.43 (0.34-0.54)	L: antineoplastic & immunimodulating agents	hormone antagonists and related agents	prostate cancer
acamprosate	Kern (62,145)	IRR= 0.45 (0.30-0.68)	N: nervous system	drugs used in addictive disorders	alcohol dependence (NMDA antagonist)
acetaminophen	¶Zang Florida (3,068)	AD: HR=0.89 (0.87-0.91)	N: nervous system	other analgesics and antipyretics	pain, fever
acetaminophen	Hu (23,245 matched pairs)	AD: HR=0.64 (0.52-0.79)			
albuterol	¶Zang MarketScan (12,471)	AD: HR=0.72 (0.71-0.73),	R: respiratory system	adrenergics for systemic use	bronchodilator/asthma, COPD
aliskiren	Zhou	D: adjusted OR=1.02 (0.98-1.07) AD: adjusted OR=0.89 (0.83-0.96)	C: cardiovascular system	other agents acting on the renin-angiotensin system	hypertension
amitriptyline	Zang MarketScan (6,184)	AD: HR=0.89 (0.86-0.94)	N: nervous system	antidepressants	tricyclic antidepressant
amlodipine	Zang MarketScan (15,627)	AD: HR=0.97 (0.94-1.00)	C: cardiovascular system	selective calcium channel blockers with mainly vascular effects	hypertension
**amoxicillin**	Zang MarketScan (14,888)	AD: HR=0.83 (0.81-0.85)	J: anti-infectives for systemic use	beta-lactam antibacterials, penicillins	antibiotic
	Hu (27,570 matched pairs)	AD: HR=0.44 (0.36-0.54)			
ampicillin	Kern (710,193)	IRR= 0.50 (0.41-0.61)	J: anti-infectives for systemic use	beta-lactam antibacterials, penicillins	antibiotic
angiotensin II receptor blockers	Reinke	OR= 0.88	C: cardiovascular system	angiotensin II receptor blockers, plain	hypertension
antihypertensives	Nakaoku	OR= 0.90 (0.88-0.93)	C: cardiovascular system	can't specify	hypertension
aspirin	Zang MarketScan (13,642)	AD: HR=0.71 (0.71-0.72)	B: blood and blood forming organs; N: nervous system	antithrombotic agents; other analgesics and antipyretics	NSAIDs
atomoxetine	Kern (407,883)	IRR= 0.69 (0.39-1.20)	N: nervous system	psychostimulants, agents used for ADHD and nootropics	ADHD (catecholamine modulator)

atorvastatin	Zang<br>(4,837 + 27,748)	AD: HR=0.74 (0.73-0.76),<br>AD: HR=0.92 (0.90-0.94)	C: cardiovascular system	lipid-modifying agents, plain	high cholesterol
**azithromycin**	Hu (27,276 matched pairs)	AD: HR=0.51 (0.42-0.62)	J: anti-infectives for systemic use	macrolides, lincosamides and streptogramins	antibiotic
	Zang MarketScan (21,604)	AD: HR=0.74 (0.71-0.77)			
benzonatate	Hu (17,534 matched pairs)	AD: HR=0.65 (0.50-0.84)	R: respiratory system	cough suppressants exc. combinations with expectorants	cough
bupropion	Zang MarketScan (15,092)	AD: HR=0.77 (0.76-0.80)	N: nervous system	antidepressants	antidepressant, nicotine dependence
carvedilol	Zang MarketScan (10,932)	AD: HR=0.94 (0.93-0.94)	C: cardiovascular system	beta blocking agents	hypertension
celecoxib	Zang MarketScan (6,344)	AD: HR=0.90 (0.88-0.92)	M: musculo-skeletal system		arthritis
cephalexin	Hu (23,061 matched pairs)	AD: HR=0.63 (0.51-0.77)	J: anti-infectives for systemic use	other beta-lactam antibacterials	antibiotic
cefdinir	Kern<br>(7,408,135)	IRR= 0.59 (0.43-0.82)	J: anti-infectives for systemic use	other beta-lactam antibacterials	antibiotic/pneumonia, strep throat, otitis
cefpodoxime	Kern<br>(245,868)	IRR= 0.28 (0.21-0.39)	J: anti-infectives for systemic use	other beta-lactam antibacterials	antibiotic
ceftriaxone	Xu-2022<br>(91,192)	AD: OR= 0.86 (0.78-0.95)	J: anti-infectives for systemic use	other beta-lactam antibacterials	antibiotic
cefuroxime[¥]	Kern<br>(3,452,799)	IRR= 0.44 (0.35-0.54)	J: anti-infectives for systemic use	other beta-lactam antibacterials	antibiotic
	Zang MarketScan (4,496)	AD: HR=0.92 (0.91-0.94)			
chlorhexidine	Zang MarketScan (4,359)	AD: HR=0.84 (0.83-0.85)	D: dermatologicals; S: sensory organs	antiseptics and disinfectants; antiinfectives	topical antiseptic
cholecalciferol	Xu-2022<br>(447,846)	AD: OR= 0.87 (0.82-0.91)	A: alimentary tract & metabolism	vit A and D, inc combinations of the two	vitamin D3
clavulanate	Hu (26,156 matched pairs)	AD: HR=0.60 (0.49-0.74)	J: anti-infectives for systemic use	Combinations of penicillins, incl. beta-lactamase inhibitors	antibiotic supp.
clindamycin	Zang MarketScan (7,851)	AD: HR=0.97 (0.96-0.99)	J: anti-infectives for systemic use	macrolides, lincosamides and streptogramins	bacterial infections
clobetasol	Zang MarketScan (6,672)	AD: HR=0.78 (0.76-0.80)	D: dermatologicals	corticosteroids, plain	eczema, psoriasis
clopidogrel	Zang MarketScan (4,381)	AD: HR=0.96 (0.96-0.96)	B: blood and blood forming organs	antithrombotic agents	blood clot prevention

clotrimazole	Zang MarketScan (5,118)	AD: HR=0.95 (0.93-0.98)	D: dermatologicals; G: genito-urinary system and sex hormones	antifungals for topical use; antiinfectives and antiseptics, exc. combinations with coticosteroids	antifungal
cough suppressants	Reinke	OR= 0.86	R: respiratory system	cough suppressants exc. combinations with expectorants	respiratory system
diazepam	Zan MarketScan (6,808)	AD: HR=0.69 (0.67-0.71)	N: nervous system	anxiolytics	anxiety
dextromethorphan	Zan MarketScan (4,012)	AD: HR=0.79 (0.77-0.80)	R: respiratory system	cough suppressants exc. combinations with expectorants	common cold, flu
diclofenac	Zang (4,174 + 4,956)	AD: HR=0.79 (0.72-0.85)<br>AD: HR=0.93 (0.91-0.95)	M: musculo-skeletal; D: dermatologicals	antiinflammatory and antirheumatic products, non-steroids; other dermatological preparations	rheumatoid arthritis; insecticides and repellents
diphenhydramine	Zang Florida (2,324)	AD: HR=0.95 (0.92-0.98)	D: dermatologicals; R: respiratory system	antipruritics, incl. antihistamines, anesthetics, etc.; antihistamines for systemic use	hay fever, cough
duloxetine	Zang MarketScan (15,760)	AD: HR=0.92 (0.87-0.96)	N: nervous system	antidepressants	SSNRIs/antidepressa nt, anxiety
**doxycycline**	Hu (21,268 matched pairs)	AD: HR=0.52 (0.41-0.66)	A: alimentary tract & metabolism; J: anti-infectives for systemic use	stomatological preparations; tetracyclines	antibiotic
	Zang MarketScan (14,612)	AD: HR=0.86 (0.82-0.90)			
dyslipidemia drugs	Nakaoku	OR=0.92 (0.89-0.95)	C: cardiovascular system	can't specify	high cholesterol, triglycerides
emtricitabine	Kern (61,916)	IRR= 0.62 (0.39-1.01)	J: anti-infectives for systemic use	direct acting antivirals	antiviral, HIV
enoxaparin	Kern (1,772,960)	IRR= 0.42 (0.31-0.57)	B: blood and blood forming organs	antithrombotic agents	anticoagulant
ergocalciferol	Zang Florida (2,801)	AD: HR=0.88 (0.83-0.93)	A: alimentary tract & metabolism	vitamin D and analogues	vitamin D deficiency
escitalopram	Zang Florida (3,068)	AD: HR=0.68 (0.61-0.75)	N: nervous system	antidepressants	SSRIs/antidepressant

exemestane	Zhou	D: adjusted OR=0.86 (0.79-0.93)<br>AD: adjusted OR=0.79 (0.69-0.89)	L: antineoplastic & immunimodulating agents	hormone antagonists and related agents	breast cancer
famotidine	Zang Florida (3,304)	AD: HR=0.95 (0.93-0.97)	A: alimentary tract & metabolism	drugs for peptic ulcer and gastro-oesophageal reflux disease (gord)	gastroesophageal reflux disease, stomach ulcers
fluconazole	Zang MarketScan (7,732)	AD: HR=0.71 (0.69-0.74)	J: anti-infectives for systemic use	antimycotics for systemic use	fungal or yeast infections
**fluticasone**	Zang (3,607+ 14,572)	AD: HR=0.92 (0.89-0.95),<br>AD: HR=0.86 (0.83-0.87)	D: dermatologicals; R: respiratory system	corticosteroids, plain; dicongestants or inhalants	corticosteroids, anti-inflammatory/asthma, skin diseases
	Hu (22,056 matched pairs)	AD: HR=0.72 (0.58-0.89)			
fluocinonide	Zang MarketScan (3,824)	AD: HR=0.90 (0.88-0.92)	D: dermatologicals	corticosteroids, plain	eczema, dermatitis, psoriasis, allergies, rash
fluzone	Zang MarketScan (10,250)	AD: HR=0.83 (0.78-0.87)	J: anti-infectives for systemic use	viral vaccines	immunization for the prevention of influenza disease
fondaparinux	Kern (81,391)	IRR= 0.38 (0.27-0.54)	B: blood and blood forming organs	antithrombotic agents	anticoagulant
furosemide	Zang MarketScan (17,550)	AD: HR=0.91 (0.88-0.93)	C: cardiovascular system	high-ceiling diuretics	diuretic/congestive heart failure, liver disease, kidney disease
gabapentin	Zang (3,664 + 23,365)	AD: HR=0.76 (0.74-0.77),<br>AD: HR=0.79 (0.77-0.81)	N: nervous system	other analgesics and antipyretics (or antispasmodic/psycholeptics/analgesic combinations)	anticonvulsants/antiepileptic
gemfibrozil	Xu-2022 (72,691)	AD: OR= 0.76 (0.70-0.84)	C: cardiovascular system	lipid-modifying agents, plain	reduction of triglycerides
hydrochlorothiazide	Zang Florida (2,699)	AD: HR=0.84 (0.76-0.91)	C: cardiovascular system	low-ceiling diuretics, thiazides	hypertension
hydrocodone	Hu (27,112 matched pairs)	AD: HR=0.66 (0.54-0.80)	R: respiratory system	cough suppressants exc. combinations with expectorants	cough, pain
hydrocortisone	Zang MarketScan (3,560)	AD: HR=0.74 (0.77-0.81)	A: alimentary tract & metabolism; D: dermatologicals	corticosteroids, plain;	swelling, redness, itching, allergic reactions
hydroxyzine	Zang MarketScan (6,244)	AD: HR=0.74 (0.71-0.77)	N: nervous system	anxiolytics	anxiety, allergies

**ibuprofen**	Xu-2022 (712,103)	AD: OR= 0.80 (0.74-0.87) HR=0.60 (0.46-0.78)	M: musculo-skeletal; G: genito-urinary system and sex hormones	anti-inflammatory and antirheumatic products, non-steroids; other gynecologicals	anti-inflammatory
	Hu (17,714 matched pairs)	AD: HR=0.60 (0.46-0.78)			
	Zang Florida (3,554) Zang MarketScan (20,731)	AD: HR=0.86 (0.83-0.89) AD: HR= 0.80 (0.78-0.82)			
ketoconazole	Zang MarketScan (6,960)	AD: HR=0.89 (0.87-0.91)	D: dermatologicals; G: genito-urinary system and sex hormones; J: anti-infectives for systemic use	antifungals for topical use; antiinfectives and antiseptics, exc. combinations with corticosteroids; antimycotics for systemic use	antifungal
letrozole	Zhou	D: adjusted OR=0.99 (0.94-1.04) AD: adjusted OR=0.89 (0.83-0.96)	L: antineoplastic & immunomodulating agents	hormone antagonists and related agents	breast cancer
levetiracetam	Kern (351,986)	IRR= 0.53 (0.42-0.66)	N: nervous system	antiepileptics	anticonvulsant
lidocaine	Zang MarketScan (8,464)	AD: HR=0.92 (0.90-0.94)	C: cardiovascular system; D: dermatologicals	antiarrhythmics, class I and III	antiarrhythmics; anaesthetics for topical use
linezolid	Kern (131,921)	IRR= 0.48 (0.37-0.62)	J: anti-infectives for systemic use	other antibacterials	antibiotic/catecholami ne modulator
lipid modifying agents	Reinke	OR= 0.90	C: cardiovascular system	lipid-modifying agents, plain	high cholesterol, triglycerides
liraglutide	Zhou	D: adjusted OR=0.80 (0.77-0.84) AD: adjusted OR=0.76 (0.70-0.82)	A: alimentary tract & metabolism	blood glucose lowering drugs, exc. insulins	T2 diabetes
lisinopril	Zang Florida (3,115)	AD: HR=0.87 (0.85-0.90)	C: cardiovascular system	ACE inhibitor	hypertension
loratadine	Zang Florida (1,777)	AD: HR=0.86 (0.85-0.88)	R: respiratory system	antihistamines for systemic use	hay fever, hives
lorcaserin	Zhou	D: adjusted OR=0.45 (0.31-0.64) AD: not ranked among top features	A: alimentary tract & metabolism	antiobesity preparations, exc. diet products	obesity
**losartan**	Zang MarketScan (19,444)	AD: HR=0.97 (0.94-0.99)	C: cardiovascular system	angiotensin II receptor blockers, plain	hypertension, heart failure, diabetes

	Hu (13,399 matched pairs)	AD: HR=0.73 (0.54-0.97)			
macrolides	Reinke	OR= 0.89	J: anti-infectives for systemic use	macrolides, lincosamides and streptogramins	antibacterials
magnesium sulfate	Hu (12,507 matched pairs)	AD: HR=0.44 (0.31-0.63)	A: alimentary tract & metabolism	drugs for constipation, other mineral supplements;	electrolyte
meloxicam	Zang Florida (2,700)	AD: HR=0.74 (0.73-0.76)	M: musculoskeletal system	anti-inflammatory and antirheumatic products, non-steroids	NSAIDs
metformin	Zang MarketScan (8,065)	AD: HR=0.96 (0.96-0.96)	A: alimentary tract & metabolism	blood glucose lowering drugs, exc.insulins	T2 diabetes
**methylprednisolone**	Zang MarketScan (10,004)	AD: HR=0.75 (0.74-0.76)	D: dermatologicals; H: systemic hormonal preparations	corticosteroids, plain; corticosteroids for systemic use, plain	anti-inflammatory
methylprednizone	Hu (24,723)	AD: HR=0.51 (0.41-0.63)	D: dermatologicals; H: systemic hormonal preparations	corticosteroids, plain; corticosteroids for systemic use, plain	anti-inflammatory
metoprolol	Zang MarketScan (17,382)	AD: HR=0.95 (0.91-0.98)	C: cardiovascular system	beta blocking agents	hypertension
methocarbamol	Zang MarketScan (3,904)	AD: HR=0.81 (0.79-0.83)	M: musculoskeletal system	muscle relaxants, centrally acting agents	
metronidazole	Zang MarketScan (4,684)	AD: HR=0.79 (0.77-0.81)	A: alimentary tract & metabolism; antiparasitic products, insecticides and repellents	stomatological preparations; agents against amoebiasis and other protozoal diseases	skin infections, rosacea, mouth infections
mirabegron	Zang MarketScan (6,096)	AD: HR=0.92 (0.91-0.92)	G: genito urinary system and sex hormones	urologicals	overactive bladder
**mirtazapine**	Kern (862,577)	IRR= 0.49 (0.43-0.55)	N: nervous system	antidepressants	antidepressant/catecholamine modulator
	Zang Florida (3,239)	AD: HR=0.83 (0.77-0.89)			
montelukast	Hu (7,254)	AD: HR=0.62 (0.42-0.91)	R: respiratory system	other systemic drugs for obstructive airway diseases	anti-inflammatory
mupirocin	Zang MarketScan (8,660)	AD: HR=0.96 (0.94-0.98)	D: dermatologicals; R: respiratory system	antibiotics for topical use; decongestants for topical use	antibiotic
	Zang Florida (2,056)	AD: HR=0.71 (0.69-0.73)			

naproxen	Zang MarketScan (7,400)	AD: HR=0.91 (0.88-0.93)	M: Musculoskeletal system	antiinflammatory and antirheumatic products, non-steroids	arthritis, joint pain
nicametate citrate	Park	AD: Among top 10 features OR=0.743	C: cardiovascular system	can't specify	vasodilator/hypertension
nystatin	Zang MarketScan (2,944)	AD: HR=0.75 (0.73-0.77)	A: alimentary tract & metabolism; D: dermatologicals	intenstinal antiinfectives; antifungals for topical use	fungal infections inside mouth
omeprazole	Zang (3,017 + 21,799)	AD: HR=0.86 (0.84-0.88), AD: HR=0.91 (0.89-0.93)	A: alimentary tract & metabolism	drugs for peptic ulcer and gastro-oesophageal reflux disease	PPIs/gastroesophageal reflux disease
oxcarbazepine	Kern (252,034)	IRR= 0.49 (0.44-0.54)	N: nervous system	antiepileptics	epilepsy
oxycodone	Zang MarketScan (8,440)	AD: HR=0.74 (0.72-0.76)	N: nervous system	opioids	severe, acute pain
palonosetron	Kern (385,832)	IRR= 0.51 (0.45-0.58)	A: alimentary tract & metabolism	Antihistamines, which are often used as antiemetics	nausea
pantoprazole	Zang (3,608 + 20,135)	AD: HR=0.81 (0.80-0.83), AD: HR=0.94 (0.92-0.96)	A: alimentary tract & metabolism	drugs for peptic ulcer and gastro-oesophageal reflux disease	PPIs/gastroesophageal reflux disease
pegfilgrastim	Kern (305,935)	IRR= 0.53 (0.41-0.70)	L: antineoplastic & immunimodulating agents	immunostimulants	colony stimulating factor
penicillin	Zang MarketScan (11,595)	AD: HR=0.89 (0.87-0.91)	J: anti-infectives for systemic use	beta-lactam antibacterials, penicillins	antibiotic
penicillin v potassium	Zang MarketScan (2,428)	AD: HR=0.93 (0.91-0.96)	J: anti-infectives for systemic use	beta-lactam antibacterials, penicillins	antibiotic
polyethylene glycol	Zang Florida (2,368)	AD: HR=0.79 (0.75-0.83)	A: alimentary tract & metabolism	drugs for constipation	laxative
**prednisone**	Zang MarketScan (10,172)	AD: HR=0.94 (0.92-0.96)	A: alimentary tract & metabolism; H: systemic hormonal preparations	intestinal anti-inflammatory agents; corticosteroids for systemic use, plain	anti-inflammatory
	Hu (26,539 matched pairs)	AD: HR=0.58 (0.48-0.71)			
pregabalin	Zan MarketScan (7,143)	AD: HR=0.53 (0.51-0.54)	N: nervous system	other analgesics and antipyretics	nerve pain
promethazine	Hu (13,467 matched pairs)	AD: HR=0.62 (0.47-0.82)	R: respiratory system; D: dermatologicals	antihistamines for systemic use; antipruritics inc. antihistamines, anaesthetics, etc.	antihistamine

pyridostigmine	Zhou	D: adjusted OR=0.89 (0.80-0.98)<br>AD: adjusted OR=0.80 (0.67-0.95)	N: nervous system	parasympathomimetics (also level 4-Anticholinesterases)	myasthenia gravis
quinidine	Kern<br>(17,789)	IRR= 0.39 (0.29-0.53)	C: cardiovascular system	antiarrhythmics, class I and III	agitation in AD
rivaroxaban	Zang MarketScan (6,796)	AD: HR=0.90 (0.89-0.90)	B: blood and blood forming organs	antithrombotic agents	vein thrombosis
ropinirole	Zang MarketScan (4,544)	AD: HR=0.95 (0.93-0.97)	N: nervous system	dopaminergic agents	Parkinson’s disease
rosuvastatin	Zhou	D: adjusted OR=1.01 (1.00-1.02)<br>AD: adjusted OR=0.97 (0.95-0.99)	C: cardiovascular system	lipid-modifying agents, plain	hyperlipidaemia
sitagliptin	Zang MarketScan (5,084)	AD: HR=0.89 (0.87-0.91)	A: alimentary tract & metabolism	blood glucose lowering drugs, exc. insulins	T2 diabetes
sertraline	Zang Florida (2,836)	AD: HR=0.83 (0.73-0.92)	N: nervous system	antidepressants	SSRIs/antidepressant
spironolactone	Zang MarketScan (8,232)	AD: HR=0.92 (0.91-0.94)	C: cardiovascular system	aldosterone antagonists and other potassium-sparing agents	hypertension,heart failure
sodium sulfate	Hu (22,539)	AD: HR=0.53 (0.41-0.69)	A: alimentary tract & metabolism	drugs for constipation	laxative
tamsulosin	Zang MarketScan (6,286)	AD: HR=0.96 (0.96-0.96)	G: genito urinary system and sex hormones	urologicals	benign prostate enlargement
tizanidine	Zang MarketScan (6,592)	AD: HR=0.73 (0.71-0.75)	M: musculo-skeletal	muscle relaxants, centrally acting agents	muscle relaxant
tolfenamic acid 200g	Park	AD: Among top 10 features OR=0.766	M: musculo-skeletal	anti-inflammatory and antirhematic products, non-steroids	NSAIDs
tramadol	Zang MarketScan (19,599)	AD: HR=0.95 (0.92-0.98)	N: nervous system	opioids	opiate analgesics
trazodone	Zang Florida (4,497)	AD: HR=0.85 (0.79-0.81)	N: nervous system	antidepressants	SARIs/antidepressant
triamcinolone	Zang MarketScan (11,388)	AD: HR=0.90 (0.88-0.91)	D: dermatologicals; R: respiratory system	corticosteroids, plain; decongestants and inhalants	anti-inflammatory
vaccines: hepatitis A, typhoid,<br>hep.A and typhoid combined, diphtheria	Wilkinson<br>(1052; 1096; 261; 1600)	Hepatitis A: HR=0.78 (p<0.001)<br>Typhoid: HR=0.80 (p<0.001)<br>Hepatitis A & typhoid combined: HR= 0.68 (p<0.001)<br>Diphtheria: HR= 0.73 (p<0.001)	J: anti-infectives for systemic use	immunoglobulins, bacterial vaccines, bacterial and viral vaccines, combined, bacterial vaccines	vaccines

valacyclovir	Hu (8,519)	AD: HR=0.56 (0.37-0.84)	J: anti-infectives for systemic use	direct acting antivirals	antiviral
valproate	Kern (553,341)	IRR= 0.36 (0.26-0.49)	N: nervous system	antiepileptics	anticonvulsant
valsartan	Zang MarketScan (4,244)	AD: HR=0.92 (0.91-0.94)	C: cardiovascular system	angiotensin II receptor blockers, plain	hypertension
varenicline	Zhou	D: adjusted OR=0.92 (0.88-0.95)<br>AD: adjusted OR=0.84 (0.78-0.91)	N: nervous system;<br>S: sensory organs	drugs used in addictive disorders; other ophthalmological	nicotine dependence
venlafaxine	Zang MarketScan (9,988)	AD: HR=0.93 (0.88-0.98)	N: nervous system	antidepressants	SNRIs/antidepressant
zolpidem	Zang MarketScan (7,100)	AD: HR=0.87 (0.86-0.89)	N: nervous system	hypnotics and sedatives	hypnotic

Note: *ATC (World Health Organisation Anatomical Therapeutic Chemical classification) is a drug classification system with five levels. ATC level 1 describes main anatomical or pharmacological groups and level 3 pharmacological subgroups; provided indications are those included in the ATC descriptions.
**Sample size per medication was not reported in all studies but it is included where available.
¶Zang et al. report results from two databases (One Florida and Market Scan) separately, therefore these are reported in the table in the same order. The sample size provided is the sum of numbers of treated and control patients whose data were used in the emulated trials.
¥potential overlap between database MarketScan used by Kern and Zang as details on versions used by the former was not clear.
Estimates are for all-cause dementia unless AD is specified; medications **in bold** reported associations in ≥2 studies.
AD: Alzheimer's disease, D: dementia, HR: hazard's ratio, IRR: incidence rate ratio, OR: odds ratio, T2 diabetes: type 2 diabetes.

Supplementary Table 4. List of medications associated with increased AD or dementia risk, ATC* level and indication					
**Medication name/group**	**study (N sample size)****	**Finding or estimate of association (95% confidence interval)**	**ATC-Level 1**	**ATC-Level 3**	**Indication**
acamprosate calciumǂ	Wilkinson	HR=4.15	N: nervous system	drugs used in addictive disorders	alcohol dependence
alendronic acid	Wilkinson	HR=1.42	M: musculoskeletal system	drugs affecting bone structure and mineralization	osteoporosis
amisulprideǂ	Wilkinson	HR=7.32	N: nervous system	antipsychotics	schizophrenia
amitriptyline hydrochloride	Wilkinson	HR=1.26	N: nervous system	antidepressants	
amoxicillin	Wilkinson	HR=1.34	J: anti-infectives for systemic use	beta-lactam antibacterials, penicillins	antibiotic
anastrozole	Zhou	D: adjusted OR=1.10 (1.06-1.14)<br>AD: adjusted OR=1.05 (1.00-1.10)	L: antineoplastic & immunimodulating agents	hormone antagonists and related agents	breast cancer
antiseptic emollients	Wilkinson	HR=1.33	D: dermatologicals		various
aripiprazole	Wilkinson	HR=6.05	N: nervous system	antipsychotics	schizophrenia
ascorbic acid	Wilkinson	HR=2.00	A: alimentary tract & metabolism	vitamin C, inc. combinations	vitamin C
aspirin (antiplatelet)<br>aspirin (musculoskeletal use)	Wilkinson	HR=1.6<br>HR=1.39	B: blood and blood forming organs	antithrombotic agents	TIA, stroke, pain
atorvastatin	Wilkinson	HR=1.16	C: cardiovascular system	lipid-modifying agents, plain	high cholesterol
baclofen	Wilkinson	HR=2.31	M: musculoskeletal system	muscle relaxants, centrally acting agents	pain, MS
betahistine hydrochloride	Wilkinson	HR=1.25			
biphasic isophane insulinǂ	Wilkinson	HR=2.2	A: alimentary tract & metabolism	insulins and analogues	diabetes
biphasic isophane insulin 2	Wilkinson	HR=2.02	A: alimentary tract & metabolism	insulins and analogues	diabetes
biphasic isophane insulin lispro	Wilkinson	HR=2.24	A: alimentary tract & metabolism	insulins and analogues	diabetes
bisacodyl	Wilkinson	HR=2.04	A: alimentary tract & metabolism	drugs for constipation	constipation

bisoprolol fumarate	Wilkinson	HR=1.21	C: cardiovascular system	beta blocking agents	hypertension, heart disease
bumetanide	Wilkinson	HR=1.60	C: cardiovascular system	high-ceiling diuretics	fluid retention caused by congestive heart failure, kidney disease
buprenorphine	Wilkinson	HR=1.60	N: nervous system	opioids	severe pain
calcium salts	Wilkinson	HR=1.40	A: alimentary tract & metabolism	mineral supplements	hypocalcemia, hyperkalemia, skin conditions
carbamazepineɫ	Wilkinson	HR=1.77	N: nervous system	antiepileptics	seizures
catheter patency solutions	Wilkinson	HR=3.44	-	-	-
cefaclor	Wilkinson	HR=1.18	J: anti-infectives for systemic use	other beta-lactam antibacterials	antibiotic
cephalexin	Nori	AD: Among top model features<br>Lasso coefficient: 0.1<br>VIF: 1.12	J: anti-infectives for systemic use	other beta-lactam antibacterials	antibiotic
	Wilkinson	HR=1.28			
chlordiazepoxide	Wilkinson	HR=1.67	N: nervous system	anxiolytics	anxiety
chlorhexidine A-H	Wilkinson	HR=1.67	A: alimentary tract & metabolism	anti-infectives and antiseptics for local oral treatment	mouth ulcer, throat infection
chlorhexidine gluconate	Wilkinson	HR=1.46	A: alimentary tract & metabolism	anti-infectives and antiseptics for local oral treatment	various
chlorpromazineɫ	Wilkinson	HR=2.95	N: nervous system	antipsychotics	schizophrenia, psychosis, agitation
cholecalciferol	Wilkinson	HR=1.61	A: alimentary tract & metabolism	vitamin D and analogues	various
cinnarizine	Wilkinson	HR=1.31	N: nervous system	antivertigo preparations	nausea
ciprofloxacin	Wilkinson	HR=1.24	J: anti-infectives for systemic use	quinolone antibacterials	UTI, STI
**citalopram**	Nori	AD: Among top model features<br>Lasso coefficient: 0.25<br>VIF: 1.06	N: nervous system	antidepressants	SSRIs/antidepressant
	Wilkinson	HR=2.64			

cleansing preparations	Wilkinson	HR=1.95			
clomipramineƗ	Wilkinson	HR=1.94	N: nervous system	antidepressants	depression, phobias
clonazepam	Wilkinson	HR=2.65	N: nervous system	antiepileptics	epilepsy
**clopidogrel** bisulfate	Nori	AD: Among top model features<br>Lasso coefficient: 0.09<br>VIF: 1.11	B: blood and blood forming organs	antithrombotic agents	stroke, heart attack
	Wilkinson	HR= 1.7			
clotrimazole<br>clotrimazole (skin)		HR=1.27<br>HR=1.23	G: genito urinary system and sex hormones<br>D: dermatologicals	anti-infectives and antiseptics, excl. combinations with corticosteroids	fungal skin infections
codeine	Wilkinson	HR=1.27	R: respiratory system	cough suppressants, excl. combinations with expectorants	pain, cough
compound analgesics x2	Wilkinson	HR range= 1.22-1.30	N: nervous system		pain
compound bronchodilators	Wilkinson	HR=1.37	R: respiratory system		
compound iron preparations	Wilkinson	HR=1.67	B: blood and blood forming organs	iron preparations	anaemia, iron deficiency
compound proprietary antacids x2	Wilkinson	HR range=1.22-1.23	A: alimentary tract & metabolism	antacids	indigestion, heartburn, acid reflux or gastro-oesophageal reflux disease
cyanocobalamin	Wilkinson	HR=2.2	B: blood and blood forming organs	vitamin B12	pernicious anaemia, malabsorption, helicobacter pylori infection
cyclizine	Wilkinson	HR=1.66	R: respiratory system	antihistamines	vertigo, motion sickness
dantronƗ	Wilkinson	HR=1.82	A: alimentary tract & metabolism	drugs for constipation	constipation in palliative care
dexamethazone	Wilkinson	HR=1.84	H: systemic hormonal preparations, excl. sex hormones and insulins	corticosteroids for systemic use, plain	blood/hormone disorders
diazepam	Wilkinson	HR=1.56	N: nervous system	anxiolytics	anxiety
digoxin	Wilkinson	HR=1.67	C: cardiovascular system	cardiac glycosides	heart failure
dihydrocodeine tartrate	Wilkinson	HR=1.3	N: nervous system	opioids	severe pain

diltiazem hydrochloride	Wilkinson	HR=1.26	C: cardiovascular system	muscle relaxants	atrial arrhythmia, hypertension, angina
dipyridamole	Wilkinson	HR=2.21	B: blood and blood forming organs	antithrombotic agents	secondary stroke prevention
dipyridamole + aspirin	Wilkinson	HR=2.20	B: blood and blood forming organs	antithrombotic agents	stroke
disulfiram	Zhou	D: adjusted OR=1.45 (1.18-1.79)<br>AD: not ranked among top features	N: nervous system; P: Antiparasitic products, insecticides and repellents	drugs used in addictive disorders; ectoparasiticides, inc. scabicides	alcohol dependence
docusate sodium	Wilkinson	HR=1.83	A: alimentary tract & metabolism	drugs for constipation	constipation
domperidone	Wilkinson	HR=1.53	A: alimentary tract & metabolism	propulsives	nausea
donepezil	Hu<br>(1,067 matched pairs)	AD: HR=30.70 (15.78-59.76)	N: nervous system	anti-dementia drugs	dementia
dosulepinł	Wilkinson	HR=1.47	N: nervous system	antidepressants	moderate to severe depression
dressings	Wilkinson	HR=1.71			not specified
**duloxetine**	Nori	AD: Among top model features<br>Lasso coefficient: 0.45<br>VIF: 1.05	N: nervous system	antidepressants	not specified
	Wilkinson	HR=2.32			
emollients (bath additives, creams) x5	Wilkinson	HR=1.25-1.85	D: dermatologicals		
eperisone	Park	OR=1.29	M: musculoskeletal;	muscle relaxants, centrally acting agents	not specified
**escitalopram**	Hu (3,830 matched pairs)	HR=3.26 (1.89-5.61)	N: nervous system	antidepressants	SSRIs/antidepressant
	Wilkinson	HR=1.84			
eszopiclone	Hu<br>(1003 matched pairs)	AD: HR=3.00 (1.09-8.28)	N: nervous system	hypnotics and sedatives	insomnia
etoricoxib	Zhou	D: adjusted OR=1.69 (1.15-2.48)<br>AD: not ranked among top features	M: musculoskeletal;	anti-inflammatory and antirheumatic products, non-steroids	osteoarthritis

exenatide	Wilkinson	HR=2.08	A: alimentary tract & metabolism	blood glucose lowering drugs exc. insulins	T2 diabetes
feed thickening preps	Wilkinson	HR=8.63			not specified
ferrous fumarate	Wilkinson	HR=1.66	B: blood and blood forming organs	iron preparations	iron deficiency anaemia
ferrous sulfate	Wilkinson	HR=1.53	B: blood and blood forming organs	iron preparations	iron deficiency anaemia
flucloxacillin	Wilkinson	HR=1.24	J: anti-infectives for systemic use	other beta-lactam antibacterials	antibiotic
fludrocortisone acetate	Wilkinson	HR=4.97	H: systemic hormonal preparations, excl. sex hormones and insulins	corticosteroids for systemic use, plain	Addison’s disease
fluoxetine	Wilkinson	HR=1.81	N: nervous system	antidepressants	depression, OCD,
flupentixolⱡ	Wilkinson	HR=1.79	N: nervous system	antipsychotics	schizophrenia, depression
fluvastatin	Zhou	D: adjusted OR=1.28 (1.20-1.36)<br>AD: adjusted OR=1.19 (1.09-1.31)	C: cardiovascular system	lipid-modifying agents, plain	high cholesterol
folic acid	Wilkinson	HR=2.74			
furosemide	Nori	AD: Among top model features<br>Lasso coefficient: 0.0<br>VIF: 1.46	C: cardiovascular system	high-ceiling diuretics	congestive heart failure, liver disease, kidney disease
	Wilkinson	HR=1.54			
fusidic acid	Wilkinson	HR=1.27	D: dermatologicals	antibiotics for topical use	antibiotic
gabapentin	Nori	AD: Among top model features<br>Lasso coefficient: 0.07<br>VIF: 1.12	N: nervous system	other analgesics and antipyretics (or Antispasmodic/psycholeptics/analgesic combinations)	anticonvulsants/antiepileptic
	Wilkinson	HR=1.53			
gemfibrozil	Zhou	D: adjusted OR=1.10 (1.08-1.13)<br>AD: adjusted OR=1.01 (0.97-1.05)	C: cardiovascular system	lipid-modifying agents, plain	reduction of triglycerides
generic expectorants	Wilkinson	HR=1.37	R: respiratory system		cold, cough
glibenclamideⱡ	Wilkinson	HR=2.05	A: alimentary tract & metabolism	blood glucose lowering drugs, exc.insulins	diabetes

gliclazide	Wilkinson	HR=1.64	A: alimentary tract & metabolism	blood glucose lowering drugs, exc.insulins	T2 diabetes
glimepiride	Wilkinson	HR=1.69	A: alimentary tract & metabolism	blood glucose lowering drugs, exc.insulins	T2 diabetes
glucagonɫ	Wilkinson	HR=2.81	H: systemic hormonal preparations, exc. sex hormones and insulins	glycogenolytic hormones	diabetic hypoglycaemia
glucose	Wilkinson	HR=2.89	A: alimentary tract & metabolism	blood glucose lowering drugs, exc.insulins	hypoglycemia
glyceryl trinitrate	Wilkinson	HR=1.27	C: cardiovascular system	vasodilators used in cardiac diseases	angina
haloperidolɫ	Wilkinson	HR=4.18	N: nervous system	antipsychotics	schizophrenia
hexetidine	Wilkinson	HR=1.74	A: alimentary tract & metabolism	stomatological preparations	antiinfective/antiseptic
hydrocodone+acetaminophen	Nori	AD: Among top model features<br>Lasso coefficient: 0.19<br>VIF: 1.27	N: nervous system	opioids	severe pain
hydrocortisone + antimicrobials	Wilkinson	HR=1.19	D: dermatologicals;<br>S: sensory organs	corticosteroids, combinations with antibiotics; anti-inflammatory agents and antiinfectives in combination	allergic ophthalmic, respiratory conditions
hydroxocobalamin	Wilkinson	HR=2.12	B: blood and blood forming organs	vitamin B12 and folic acid	vitamin B12 deficiency anaemia
hyoscine hydrobromide (CNS use)	Wilkinson	HR=2.88			nausea, motion sickness
imipramineɫ	Wilkinson	HR=1.81	N: nervous system	antidepressants	depression
influenza vaccines	Wilkinson	HR=1.14	J: anti-infectives for systemic use	viral vaccines	influenza
insulin aspart	Wilkinson	HR=2.18	A: alimentary tract & metabolism	insulins and analogues	diabetes
insulin glargine	Wilkinson	HR=2.18	A: alimentary tract & metabolism	insulins and analogues	diabetes
iodine compounds	Wilkinson	HR=1.54			
ipratropium bromide	Wilkinson	HR=1.34	R: respiratory system	decongestants and other nasal preparations for topical use	COPD, bronchitis
isophane insulinɫ	Wilkinson	HR=2.58	A: alimentary tract & metabolism	insulins and analogues	diabetes

isosorbide mononitrate	Wilkinson	HR=1.39	C: cardiovascular system	vasodilators used in cardiac diseases	angina, heart failure
ispaghula husk	Wilkinson	HR=1.39	A: alimentary tract & metabolism	drugs for constipation	constipation
lactulose	Wilkinson	HR=1.80	A: alimentary tract & metabolism	drugs for constipation	constipation
lamotrigineɫ	Wilkinson	HR=3.79	N: nervous system	antiepileptics	seizures
lansoprazole	Wilkinson	HR=1.20	A: alimentary tract & metabolism	drugs for peptic ulcer and gastro-oesophageal reflux disease	peptic ulcer and gastro-oesophageal reflux disease
**levetiracetam**	Hu (877 matched pairs)	AD: HR=4.03 (1.51-10.75)	N: nervous system	antiepileptics	seizures
	Wilkinson	HR=4.99			
levodopa	Hu (727 matched pairs)	AD: HR=3.44 (1.27-9.31)	N: nervous system	dopaminergic agents	Parkinson's disease
levodopa with benserazideɫ	Wilkinson	HR=8.55	N: nervous system	dopaminergic agents	Parkinson's disease
levodopa with carbidopaɫ	Wilkinson	HR=7.83	N: nervous system	dopaminergic agents	Parkinson's disease
linagliptin	Wilkinson	HR=1.89	A: alimentary tract & metabolism	blood glucose lowering drugs, exc.insulins	T2 diabetes
lisinopril	Nori	AD: Among top model features<br>Lasso coefficient: 0.11<br>VIF: 1.17	C: cardiovascular system	ACE inhibitor	hypertension
lithium carbonateɫ	Wilkinson	HR=3.59	N: nervous system	antipsychotics	mania, bipolar disorder
local anaesthetics (skin)	Wilkinson	HR=1.46	S: sensory organs	local anaesthetics	various
lofepramineɫ	Wilkinson	HR=1.69	N: nervous system	antidepressants	depression
loperamide hydrochloride	Wilkinson	HR=1.42	A: alimentary tract & metabolism	antipropulsives	diarrhea
lorazepam	Wilkinson	HR=3.37	N: nervous system	anxiolytics	anxiety, insomnia
magnesium hydroxide	Wilkinson	HR=2.27	A: alimentary tract & metabolism; G: genito urinary system and sex hormones	antacids; urologicals	laxative
magnesium salts	Wilkinson	HR=1.63	A: alimentary tract & metabolism	antacids	heartburn
malathion	Wilkinson	HR=1.45	P: Antiparasitic products, insecticides and repellents	ectoparasiticides, inc. scabicides	lice, scabies

melatonin	Wilkinson	HR=2.98	N: nervous system	hypnotics and sedatives	sleep disorders
memantine	Hu (544 matched pairs)	HR=38.97 (14.41-105.39)	N: nervous system	anti-dementia drugs	dementia
**metformin**	Nori	AD: Among top model features<br>Lasso coefficient: 0.13<br>VIF: 1.40	A: alimentary tract & metabolism	blood glucose lowering drugs, exc.insulins	T2 diabetes
	Wilkinson	HR=1.47			
metoclopramide hydrochloride	Wilkinson	HR=1.35	A: alimentary tract & metabolism	propulsives	nausea, vomiting
metronidazole	Wilkinson	HR=1.20	A: alimentary tract & metabolism; J: anti-infectives for systemic use	stomatological preparations; other antibacterials	antibiotic
miconazole x2	Wilkinson	HR range=1.29-1.39	A: alimentary tract & metabolism; D: dermatologicals	stomatological preparations; antifungals for topical use	antifungal
minor skin infection preparations	Wilkinson	HR=1.48	D: dermatologicals		
**mirtazapine**	Hu	AD: HR=3.11 (1.70-5.68)	N: nervous system	antidepressants	antidepressant/catecholamine modulator
	Wilkinson	HR=2.95			
more supplements and complete foods x11	Wilkinson	HR= range 2.18-3.88			
morphine salts x2	Wilkinson	HR range=1.59-1.75	N: nervous system	opioids	moderate to severe pain
multivitamins	Wilkinson	HR=2.28	A: alimentary tract & metabolism	vitamins	
mupirocin	Wilkinson	HR=1.29	D: dermatologicals<br>R: respiratory system	antibiotics for topical use; decongestants and other nasal preparations for topical use	impetigo
neostigmine	Zhou	D: adjusted OR=1.42 (1.17-1.73)<br>AD: not ranked among top features	N: nervous system; S:sensory organs	parasympathomimetics (also level 4- Anticholinesterases); antiglaucoma preparations and myotics	myasthenia gravis
nicorandil	Wilkinson	HR=1.34	C: cardiovascular system	vasodilators used in cardiac diseases	angina
nicotine	Wilkinson	HR=1.37	N: nervous system	drugs used in addictive disorders	smoking cessation

nitrazepamł	Wilkinson	HR=1.59	N: nervous system	hypnotics and sedatives	insomnia
nitrofurantoin	Wilkinson	HR=1.49	J: anti-infectives for systemic use	other antibacterials	UTIs
**not specified (anti-dementia drugs)**	Xu-2020	AD: Among top features F-score: 40	N: nervous system	anti-dementia drugs	dementia
	Reinke (1,639)	OR=2.14			
not specified	Xu-2020	AD: Among top features F-score: 11	N: nervous system	antiepileptics	
not specified	Xu-2020	AD: Among top features F-score: 18	C: cardiovascular system	angiotensin II receptor blockers, plain	
**not specified (antipsychotics)**	Xu-2020	AD: Among top features F-score: 29	N: nervous system	antipsychotics	
	Reinke (5,827)	OR=1.64			
	Nakaoku	OR= 1.40 (1.31-1.49)			
not specified	Xu-2020	AD: Among top features F-score: 20	R: respiratory system	adrenergics, inhalants	
not specified	Xu-2020	AD: Among top features F-score: 14	N: nervous system	dopaminergic agents	
not specified	Xu-2020	AD: Among top features F-score: 7	C: cardiovascular system	vasodilators	
not specified	Xu-2020	AD: Among top features F-score: 11	? V: Various	anti-inflammatory agents	
not specified	Xu-2020	AD: Among top features F-score: 6	A: alimentary tract & metabolism	other mineral supplements	
not specified	Xu-2020	AD: Among top features F-score: 10	J: anti-infectives for systemic use	direct acting antivirals	
not specified	Xu-2020	AD: Among top features F-score: 13	C: cardiovascular system	beta blocking agents	
**not specified (antidepressants)**	Xu-2020	AD: Among top features F-score: 19	N: nervous system	antidepressants	
	Reinke (14,601)	OR=1.21			
	Nakaoku	OR= 1.38 (1.31-1.44)			
	Miled	Among top features			
not specified	Xu-2020	AD: Among top features F-score: 5	A: alimentary tract & metabolism	vit 12 and folic acid	
not specified	Reinke	OR=1.39	N: nervous system	psychostimulants	
not specified	Reinke	OR=1.21	G: genito-urinary system and sex hormones	urologicals	

not specified	Reinke	OR=1.25	A: alimentary tract & metabolism	insulins and analogues	
not specified	Nakaoku	OR= 1.17 (1.11-1.24)	N: nervous system	hypnotics	
not specified	Nakaoku	OR= 1.06 (1.02-1.10)	B: blood and blood forming organs	antithrombotic agents	
not specified	Fukunishi	AD: Among top features		anti-inflammatory agents	
not specified	Fukunishi	AD: Among top features		analgesics	
not specified	Miled	Among top features	C: cardiovascular system	diuretics	
not specified	Miled	Among top features	C: cardiovascular system	antihyperlipidemics	
not specified	Miled	Among top features	C: cardiovascular system	antihypertensives	
not specified	Miled	Among top features	N: nervous system	opioid analgesics	
not specified	Miled	Among top features	N: nervous system	psychotherapeutic and neurological agents	
nystatin x2	Wilkinson	HR range=1.39-1.41	A: alimentary tract & metabolism; D: dermatologicals	intenstinal antiinfectives; antifungals for topical use	fungal infections inside mouth
olanzapineł	Wilkinson	HR=5.14	N: nervous system	antipsychotics	mania, schizophrenia
omeprazole	Wilkinson	HR=1.21	A: alimentary tract & metabolism	drugs for peptic ulcer and gastro-oesophageal reflux disease	ulcer and gastro-oesophageal reflux disease
other oropharyngeal preparations	Wilkinson	HR=1.86			
**oxybutynin**	Nori	AD: Among top model features<br>Lasso coefficient: 0.22<br>VIF: 1.08	G: genito-urinary system and sex hormones	urologicals	urinary incontinence
	Wilkinson	HR=1.89			
oxycodone hydrochloride	Wilkinson	HR=1.60	N: nervous system	opioids	severe, acute pain
paracetamol	Wilkinson	HR=1.71	N: nervous system	other analgesics and antipyretics	mild to moderate pain
paroxetineł	Wilkinson	HR=1.61	N: nervous system	antidepressants	depression, anxiety
pericyazine	Wilkinson	HR=6.05	N: nervous system	antipsychotics	psychosis, agitation
permethrin	Wilkinson	HR=1.33	P: antiparasitic products, insecticides and repellents	ectoparasiticides, incl. scabicides	scabies
phenytoinł	Wilkinson	HR=2.79	N: nervous system	antiepileptics	seizures

pioglitazone	Zhou	D: adjusted OR=1.09 (1.08-1.11)<br>AD: adjusted OR=1.14 (1.11-1.17)	A: alimentary tract & metabolism	blood glucose lowering drugs, exc.insulins	T2 diabetes
polyethylene glycol electrolyte	Wilkinson	HR=2.06			colonoscopy, colon surgery
potassium chloride	Nori	AD: Among top model features<br>Lasso coefficient: 0.24<br>VIF: 1.35	A: alimentary tract & metabolism	mineral supplements	hypokalaemia
potassium salts	Wilkinson	HR=1.69	B: blood and blood forming organs	solution additives	
potassium sparing compound diuretics a-z	Wilkinson	HR=1.3	B: blood and blood forming organs	solution additives	
pramipexole	Wilkinson	HR=2.86	N: nervous system	dopaminergic agents	Parkinson's disease
pravastatin	Zhou	D: adjusted OR=1.35 (1.34-1.36)<br>AD: adjusted OR=1.28 (1.26-1.29)	C: cardiovascular system	lipid-modifying agents, plain	high cholesterol
prednisone	Zhou	D: adjusted OR=1.15 (1.12-1.17)<br>AD: adjusted OR=1.01 (0.97-1.04)	A: alimentary tract & metabolism; H: systemic hormonal preparations	intestinal anti-inflammatory agents; corticosteroids for systemic use, plain	anti-inflammatory
pregabalin	Wilkinson	HR=1.56	N: nervous system	other analgesics and antipyretics	neuropathic pain
primidoneł	Wilkinson	HR=2.53	N: nervous system	antiepileptics	essential tremor, epilespy
prochlorperazine	Wilkinson	HR=1.20	N: nervous system	antipsychotics	nausea
procyclidine	Wilkinson	HR=4.7	N: nervous system	anticholinergic agents	parkinsonism
promazine	Wilkinson	HR=4.37	N: nervous system	antipsychotics	agitation
promethazine hydrochloride	Wilkinson	HR=1.47	R: respiratory system	antihistamines for systemic use	allergies
propoxyphene/acetaminophen	Nori	AD: Among top model features<br>Lasso coefficient: 0.15<br>VIF: 1.09	N: nervous system	opioids	mild to moderate pain
propranolol	Wilkinson	HR=1.29	C: cardiovascular system	beta blocking agents	
proprietary cough compound preparations m-z	Wilkinson	HR=1.27	R: respiratory system		

**quetiapine**	Hu (1,004 matched pairs)	AD: HR=7.19 (3.58-14.46)	N: nervous system	antipsychotics	antipsychotics
	Wilkinson	HR=7.29			
quinine	Wilkinson	HR=1.23	P: antiparasitic products, insecticides and repellents	antimalarials	malaria
ranitidine	Wilkinson	HR=1.19	A: alimentary tract & metabolism	drugs for peptic ulcer and gastro-oesophageal reflux disease	peptic ulcer and gastro-oesophageal reflux disease
rasagiline	Wilkinson	HR=7.72	N: nervous system	dopaminergic agents	Parkinson’s disease
reboxetineɫ	Wilkinson	HR=2.03	N: nervous system	antidepressants	depression
rectal laxatives	Wilkinson	HR=2.22			
renal failure/ketogen food	Wilkinson	HR=3.97	A: alimentary tract & metabolism		
risperidoneɫ	Wilkinson	HR=6.54	N: nervous system	antipsychotics	mania, schizophrenia
rivaroxaban	Wilkinson	HR=2.08	B: blood and blood forming organs	antithrombotic agents	vein thrombosis
ropinirole	Wilkinson	HR=3.65	N: nervous system	dopaminergic agents	Parkinson's disease
rosiglitazone	Wilkinson	HR=1.52	A: alimentary tract & metabolism	blood glucose lowering drugs, exc.insulins	T2 diabetes
salbutamol (inhalation preparation)	Wilkinson	HR=1.15	R: respiratory system	adrenergics, inhalants	asthma, COPD
salmeterol + fluticasone propionate	Wilkinson	HR=1.19	R: respiratory system	adrenergics, inhalants	asthma
senna	Wilkinson	HR=2.16	A: alimentary tract & metabolism	drugs for constipation	constipation
**sertraline**	Hu (4,710 matched pairs)	AD: HR=2.39 (1.67-3.42)	N: nervous system	antidepressants	SSRIs/antidepressant
	Nori	AD: Among top model features<br>Lasso coefficient: 0.29<br>VIF: 1.05			
	Wilkinson	HR=2.85			
**simvastatin**	Nori	AD: Among top model features<br>Lasso coefficient: 0.1<br>VIF: 1.17	C: cardiovascular system	lipid-modifying agents, plain	high cholesterol
	Wilkinson	HR=1.22			

sodium chloride (skin)	Wilkinson	HR=1.65	D: dermatologicals	preparations for treatment of wounds and ulcers	
sodium picosulfate	Wilkinson	HR=1.88	A: alimentary tract & metabolism	drugs for constipation	constipation
sodium salts	Wilkinson	HR=1.41	-	-	-
sodium valproateⱡ	Wilkinson	HR=4.17	N: nervous system	antiepileptics	epilepsy
solifenacin	Wilkinson	HR=2.10			
soluble neutral insulinⱡ	Wilkinson	HR=2.33	A: alimentary tract & metabolism	insulins and analogues	diabetes
spironolactone	Wilkinson	HR=1.45	C: cardiovascular system	aldosterone antagonists and other potassium-sparing agents	hypertension heart failure
**sulfamethoxazole**	Zhou	D: adjusted OR=1.37 (1.36-1.39)<br>AD: adjusted OR=1.32 (1.30-1.34)	J: anti-infectives for systemic use	sulfonamideds and trimethoprim	UTIs, otitis, etc.
	Nori	AD: Among top model features<br>Lasso coefficient: 0.15<br>VIF: 1.12			
supplements & compound feeds	Wilkinson	HR=3.12	-	-	-
tamsulosin	Wilkinson	HR=1.38	G: genito-urinary system and sex hormones	drugs used in benign prostatic hypertrophy	enlarged prostate
temazepam	Wilkinson	HR=1.61	N: nervous system	hypnotics and sedatives	insomnia
thiamine	Wilkinson	HR=5.64	A: alimentary tract & metabolism	vitamins	vitamin B1
thioridazineⱡ	Wilkinson	HR=2.59	N: nervous system	antipsychotics	schizophrenia
tiotropium	Wilkinson	HR=1.24	R: respiratory system	other drugs for obstructive airway diseases, inhalants	COPD, asthma
tolterodine tartrate	Nori	AD: Among top model features<br>Lasso coefficient: 0.32<br>VIF: 1.07	G: genito-urinary system and sex hormones	urologicals	urinary incontinence
	Wilkinson	HR=1.66			
topical anti-inflammatory preparations x3	Wilkinson	HR range= 1.19-1.22	-	-	-
**tramadol**	Nori	AD: Among top model features	N: nervous system	opioids	opiate analgesics

		Lasso coefficient: 0.09<br>VIF: 1.13			
	Wilkinson	HR=1.26			
**trazodone**	Hu (5,524 matched pairs)	AD: HR=2.37 (1.63-3.45)	N: nervous system	antidepressants	SARIs/antidepressant
	Wilkinson	HR=2.10			
trifluoperazineł	Wilkinson	HR=3.06	N: nervous system	antipsychotics	nausea, schizophrenia
trimethoprim	Wilkinson	HR=1.42	J: anti-infectives for systemic use	sulfonamides and trimethoprim	antibiotic, cystitis
trospium chloride	Wilkinson	HR=1.93	G: genito-urinary system and sex hormones	urologicals	overactive bladder
vehicles & diluents x2	Wilkinson	HR range=1.36-1.48	-	-	-
**venlafaxineł**	Nori	AD: Among top model features<br>Lasso coefficient: 0.52<br>VIF: 1.02	N: nervous system	antidepressants	SNRIs/antidepressant
	Wilkinson	HR=2.28			
vitamin B complex	Wilkinson	HR=2.94	A: alimentary tract & metabolism	vitamins B complex	vitamins
warfarin sodium	Wilkinson	HR=1.35	B: blood and blood forming organs	antithrombotic agents	prevent/treat blood clots
zolpidem	Wilkinson	HR=1.42	N: nervous system	hypnotics and sedatives	insomnia
zopiclone	Wilkinson	HR=1.66	N: nervous system	hypnotics and sedatives	insomnia
zotepine	Park	AD: OR=1.353	N: nervous system	antipsychotics	antipsychotics

Note: *ATC (World Health Organisation Anatomical Therapeutic Chemical classification) is a drug classification system with five levels. ATC level 1 describes main anatomical or pharmacological groups and level 3 pharmacological subgroups; provided indications are those included in the ATC descriptions;
**Sample size per medication was not reported in all studies but it is included where available;
ł indicates medications where associations with increased risk remained significant for all analyses in Wilkinson et al. including overall HR, 5- and 10-year sensitivity analysis;
Estimates are for all-cause dementia unless AD is specified; medications **in bold** reported associations in ≥2 studies;
AD: Alzheimer's disease, D: dementia, HR: hazard's ratio, OR: odds ratio, T2 diabetes: type 2 diabetes.
